# A second generation genetic map of the bumblebee *Bombus terrestris *(Linnaeus, 1758) reveals slow genome and chromosome evolution in the Apidae

**DOI:** 10.1186/1471-2164-12-48

**Published:** 2011-01-19

**Authors:** Eckart Stolle, Lena Wilfert, Regula Schmid-Hempel, Paul Schmid-Hempel, Michael Kube, Richard Reinhardt, Robin FA Moritz

**Affiliations:** 1 Institut für Biologie, Martin-Luther-Universität Halle-Wittenberg, Hoher Weg 4, D-06099 Halle (Saale), Germany; 2 Institute of Integrative Biology (IBZ), ETH Zürich, Universitätsstrasse 16, CH-8092 Zürich, Switzerland; 3 Max Planck Institute for Molecular Genetics, Ihnestraße 63-73, D-14195 Berlin, Germany; 4 Department of Genetics, University of Cambridge, Cambridge, CB2 3EH, UK; 5 Genome Centre Cologne at MPI for Plant Breeding Research, Carl-von-Linné-Weg 10, D-50829 Köln, Germany

## Abstract

**Background:**

The bumblebee *Bombus terrestris *is an ecologically and economically important pollinator and has become an important biological model system. To study fundamental evolutionary questions at the genomic level, a high resolution genetic linkage map is an essential tool for analyses ranging from quantitative trait loci (QTL) mapping to genome assembly and comparative genomics. We here present a saturated linkage map and match it with the *Apis mellifera *genome using homologous markers. This genome-wide comparison allows insights into structural conservations and rearrangements and thus the evolution on a chromosomal level.

**Results:**

The high density linkage map covers ~ 93% of the *B. terrestris *genome on 18 linkage groups (LGs) and has a length of 2'047 cM with an average marker distance of 4.02 cM. Based on a genome size of ~ 430 Mb, the recombination rate estimate is 4.76 cM/Mb. Sequence homologies of 242 homologous markers allowed to match 15 *B. terrestris *with *A. mellifera *LGs, five of them as composites. Comparing marker orders between both genomes we detect over 14% of the genome to be organized in synteny and 21% in rearranged blocks on the same homologous LG.

**Conclusions:**

This study demonstrates that, despite the very high recombination rates of both *A. mellifera *and *B. terrestris *and a long divergence time of about 100 million years, the genomes' genetic architecture is highly conserved. This reflects a slow genome evolution in these bees. We show that data on genome organization and conserved molecular markers can be used as a powerful tool for comparative genomics and evolutionary studies, opening up new avenues of research in the Apidae.

## Background

The buff-tailed bumblebee *Bombus terrestris *is a key pollinator for crops and wild flowering plants as well as a model system in various disciplines of biological research. This includes studies on population genetics, mating biology, sexual selection, caste determination, social behavior, host-parasite interactions, immunology and plant-pollinator interactions [1-11]. In addition, colonies of *B. terrestris *are commercially produced in large numbers in Europe for pollination of greenhouse crops [1]. Accordingly, many genomic resources have been developed for this species such as molecular markers [12-15], genetic linkage maps [16,17] and BAC- and EST-libraries [18,19].

With the advance of genome sequencing techniques *B. terrestris *is about to evolve into an important Hymenopteran genetic model species in addition to the honeybee, *Apis mellifera *and the parasitic wasp *Nasonia *spp. Since the bumblebee is phylogenetically very similar to *A. mellifera *with its fully sequenced genome, a genomic comparison between the two species is particularly rewarding for understanding genome evolution in social bees. The genome of *A. mellifera *revealed several exceptional traits including an extremely high recombination rate, a very high AT-content, the lack of retrotransposons, and a high density of simple-sequence-repeats (SSR/microsatellites) [20]. The evolution of these extraordinary genome characteristics is unclear. A comparison with the bumblebee genome might therefore reveal common patterns resulting from the phylogenetically close relationship, but also differences due to different social colony structures and ecologies of honeybees and bumblebees.

High resolution genetic maps are powerful tools to study genomic organization [21,22]. Moreover, such maps greatly facilitate genome assembly for full genome sequencing [23]. Whereas most of the first genetic maps were based on markers like RAPD, AFLP, isozymes or mutant phenotypes, linkage maps are now increasingly constructed with polymorphic simple sequence repeats (SSR, microsatellites) or single nucleotide polymorphisms (SNP) [23-35]. Since these markers also include sequence information of potentially conserved flanking regions, they allow for anchoring genome assemblies and for comparisons among species [24,28,32,35-37].

For *B. terrestris *two basic linkage maps are available: one map based on RAPD and SSR markers [16] and another map with AFLP and SSR markers [17,38]. However, in both maps the coverage and marker density was insufficient to explicitly detect all known 18 chromosomes of this species' haploid set [39]. Moreover, these maps could not be used for genomic comparisons between the honeybee and the bumblebee, because most markers were either RAPDs or AFLPs, which do not provide any sequence information.

In this paper we construct a dense and saturated genetic (meiotic) linkage map for the bumblebee *B. terrestris *using recently published SSR markers [15] as well as novel SSRs created from BAC-end sequences. Based on this second generation linkage map and sequence homologies of microsatellite-flanking regions, we compare the genetic maps of *B. terrestris *and *A. mellifera *to identify homologous chromosomes, conserved synteny blocks and rearrangements. These can be used to study chromosome and genome evolution as well as QTL synteny among species.

## Results

### SSR markers

A screen of the BAC library [19] yielded 4'593 SSRs with motifs of 1-6 bp in length of which 2'573 (56%) were redundant or had too short sequences that were flanking the repeat motif. For the remaining 2'020 loci, a total of 960 primer pairs were tested for amplification products. 910 of those (95%) yielded PCR products and were screened for polymorphisms in *B. terrestris*. 586 primer pairs (64.4%) showed two or more alleles of which 564 were tested for polymorphism in the mapping population "BBM1" [17], a subset of 300 loci by using fluorescent labels, 264 loci by using unlabeled primers. This resulted in a total of 306 informative loci. The 123 SSR loci published in ref. [15] yielded 56 additional polymorphic loci in the population BBM1 and further three novel loci were developed as described in [15]. A screen of 2'304 *A. mellifera *SSR markers [23,40-42] yielded 15 loci that were polymorphic in BBM1. (Additional file 1). Finally 274 SSRs were successfully or sufficiently genotyped.

### Map

To construct the new linkage map, we used the raw data (207 AFLPs, 39 SSRs) from the mapping population BBM1 (which was used for the core linkage map [17]) plus another 46 SSRs from ref. [15] and 209 SSRs derived from the BAC library (Additional file 2). Additionally three novel markers and 15 *Apis mellifera *[23,40-42] SSR markers were mapped (Table 1, Additional file 2). Four AFLPs remained unmapped. Although 75 markers showed segregation distortion, they were nevertheless included because their exclusion did not alter the map (Table 1).

**Table 1:** Summary of the *B. terrestris *linkage map

LG	length (cM)	markers (n)	distorted markers (n)	avg. marker distance (cM)	corrected length (cM)	**LG as in **[17,38]
***Bt.*B01**	121.01	38	5	3.18	127.38	8

***Bt.*B02**	125.20	37	6	3.38	131.97	1

***Bt.*B03**	96.35	38	1	2.54	101.42	4

***Bt.*B04**	80.66	20	1	4.03	88.73	13, BB1_18

***Bt.*B05**	102.84	30	5	3.43	109.69	11

***Bt.*B06**	171.70	35	6	4.91	181.51	BB1_15

***Bt.*B07**	161.43	33	0	4.89	171.21	9

***Bt.*B08**	91.64	30	5	3.05	97.75	2

***Bt.*B09**	109.48	32	6	3.42	116.32	5

***Bt.*B10**	126.46	35	9	3.61	133.69	12

***Bt.*B11**	116.30	35	2	3.32	122.94	7

***Bt.*B12**	111.39	34	3	3.28	117.94	3

***Bt.*B13**	105.74	31	5	3.41	112.56	6

***Bt.*B14**	73.44	21	4	3.50	80.43	BB1_16

***Bt.*B15**	96.55	33	13	2.93	102.40	10

***Bt.*B16**	77.87	16	1	4.87	87.61	BB1_17, BB1_20

***Bt.*B17**	83.14	10	2	8.31	99.77	14

***Bt.*B18**	51.01	8	1	6.38	63.77	BB1_19

**∑/ø**	**1902.21**	**516**	**75**	**4.02 ± 1.42**	**2047.09**	

For each Linkage Group (LG) the length in cM (∑), the number of markers (SSR and AFLP) mapped on this LG (∑), the number of markers showing segregation distortion (∑), the average distance between two markers in cM, the length in cM of the LG after correction for chromosome ends (∑), and the corresponding LG in [17,38] are given. At the bottom the sums and the average marker distance ± standard deviation is given, respectively.

Processing all available genotype data in JoinMap4 [43] yielded 18 linkage groups (LGs) all of which were well supported by LOD scores of 8.0 or higher (Additional file 3). The 18 LGs, which most likely represent the 18 haploid chromosomes [39], range in recombination size from 51.01 to 171.7 cM containing 8 to 38 markers (Table 1). The shortest one, LG B18, contains only five AFLP and three SSR markers, the longest LG (B06) has 35 markers. The length of a LG was correlated with the number of markers per linkage group (Pearson *r *= 0.71768, *p *< 0.05). The average marker distance ranges from 2.54 cM (LG B03) to 8.31 cM (LG B17) with an average of 4.02 cM (± 1.42 cM SD).

This map contains a total 516 markers and spans a total of 1'902.21 cM (Additional file 2, Additional file 3). This is an increase of 271.21 cM (16.62%) compared to [17] (1'630.9 cM, reanalyzed with JoinMap4 [43]). To correct for the missing chromosome ends, which cannot be mapped since there are no flanking markers, the length of each LG was adjusted by adding double its average marker distance to the value calculated by JoinMap [44]. This resulted in a corrected map length of 2'047.09 cM (Table 1). Hence the genome coverage of the present map is estimated to be 92.92%.

Based on the function c = 1-e^-2md/L ^given in ref. [45] where c is the proportion of the genome within d cM distance to a marker, L the estimated genome length and m the number of markers, 86.85% of the genome is located within the average marker distance of 4.02 cM and 99.99% of the genome is located within 17.6 cM distance to a marker.

### Genome size and recombination rate

The genome size of the bumblebee *B. terrestris *previously was measured by flow cytometry [16,17]. The first measurement [16] was based on a staining method biased towards the AT portion of the genome, hence a correction is needed. The genomic AT-content of *B. terrestris *was estimated to be 61% by using 8.5 Mb non-redundant sequences (data not shown) from the BAC library, representing about 1.98% of the genome. The honeybee AT-content is 67.3% [46], 6.3% higher than the bumblebee. Consequently the DNA content (0.27 pg) as measured by ref. [16,47] was corrected leading to an increase of the ratio (*B. terrestris*/*A. mellifera *DNA content) from 1.54 to 1.653. Thus the genome size of the bumblebee *Bombus terrestris *was estimated to be 433 Mb.

A second estimate was obtained using the relation between genetic distance and physical distance for two markers from the two ends of a BAC clone [19]. The two markers SSR_0929_66j14 and SSR_924_66j14 are 0.494 cM apart (Additional file 2). The average insert size of clones from the BAC library is 102.9 kb, based on a selection of n = 186 clones which doesn't include this BAC clone [19]. Extrapolated onto the whole map, a genome size of about 426 Mb is calculated. This nearly matches the previous estimate of 433 Mb. The average between both estimates is 430 Mb. However, preliminary data for the genome assembly of *B. terrestris *(Baylor College of Medicine Human Genome Sequencing Center, unpublished) give an additional estimate of about 250 Mb for the size of the genome.

Using the length of this linkage map (2'047.09 cM), a recombination rate of 4.76 cM/Mb is calculated, based on a genome size of 430 Mb, and 8.19 cM/Mb based on a genome size of 250 Mb.

### Homology

A search for homologous sequences in the *A. mellifera *genome for each mapped SSR marker yielded 242 homologous loci, with 15 being homologous with unassigned (unmapped) *A. mellifera *sequences. In 29 cases the *B. terrestris *sequence was homologous to a gene or a predicted gene in *A. mellifera *(Table 2, Additional file 1, Additional file 2).

**Table2:** Matching linkage groups between *B. terrestris *and *A. mellifera*

LG	*Am*. LG01	*Am*. LG02	*Am*. LG03	*Am*. LG04	*Am*. LG05	*Am*. LG06	*Am*. LG07	*Am*. LG08	*Am*. LG09	*Am*. LG10	*Am*. LG11	*Am*. LG12	*Am*. LG13	*Am*. LG14	*Am*. LG15	*Am*. LG16	*Am*. Un	∑
***Bt.*B01**	**26**			1	1													**28**

***Bt.*B02**		**17**															2	**19**

***Bt.*B03**	1		**12**					1		1						1	1	**17**

***Bt.*B04**			1	**4**														**5**

***Bt.*B05**					**19**		1											**20**

***Bt.*B06**	2		1			**14**						1					1	**19**

***Bt.*B07**	1						**3**				2						2	**8**

***Bt.*B08**							**4**	**7**									1	**12**

***Bt.*B09**	**4**					**5**			**12**									**21**

***Bt.*B10**							1			**8**							1	**10**

***Bt.*B11**	**6**				1						**7**						2	**16**

***Bt.*B12**												**8**				**7**	1	**16**

***Bt.*B13**						1							**16**				2	**19**

***Bt.*B14**				1										**10**				**11**

***Bt.*B15**							1					**7**			**6**			**14**

***Bt.*B16**	1							2									2	**5**

***Bt.*B17**					1													**1**

***Bt.*B18**																1		**1**

∑	**41**	**17**	**14**	**6**	**22**	**20**	**10**	**10**	**12**	**9**	**9**	**16**	**16**	**10**	**6**	**9**	**15**	**242**

The numbers of markers homologous between *Bombus terrestris *and *Apis mellifera *are shown for each LG. Bold numbers indicate matching *B. terrestris *and *A. mellifera *LGs, as homologized LGs (the majority of the homologous markers is found in one LG) or as composites if a LG consists of a high proportion of markers homologous to more than one *A. mellifera *LG. Single markers or low numbers of markers are left normal.

**Figure 1:**
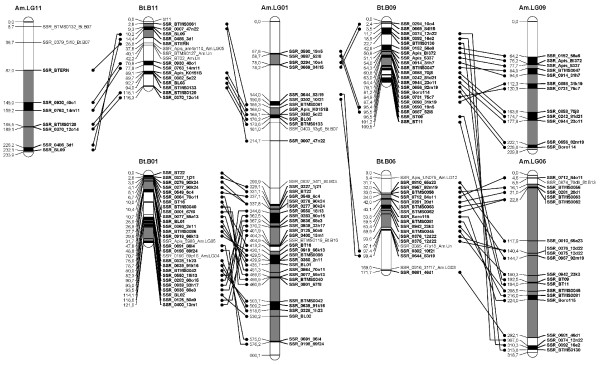
**Comparison between matched *Bombus terrestris *and *Apis mellifera *LGs**. This figure shows the homology between *B. terrestris *and *A. mellifera *LGs 1, 6, 9 and 11. Homologous linkage groups of both species are presented next to each other. Bold marker names and connecting lines indicate homologous markers. Black symbolizes synteny; grey indicates intervals between markers, which can be found in the other genome on the same (matched) LG, but rearranged; white intervals are unknown or not syntenic or homologous.

**Figure 2:**
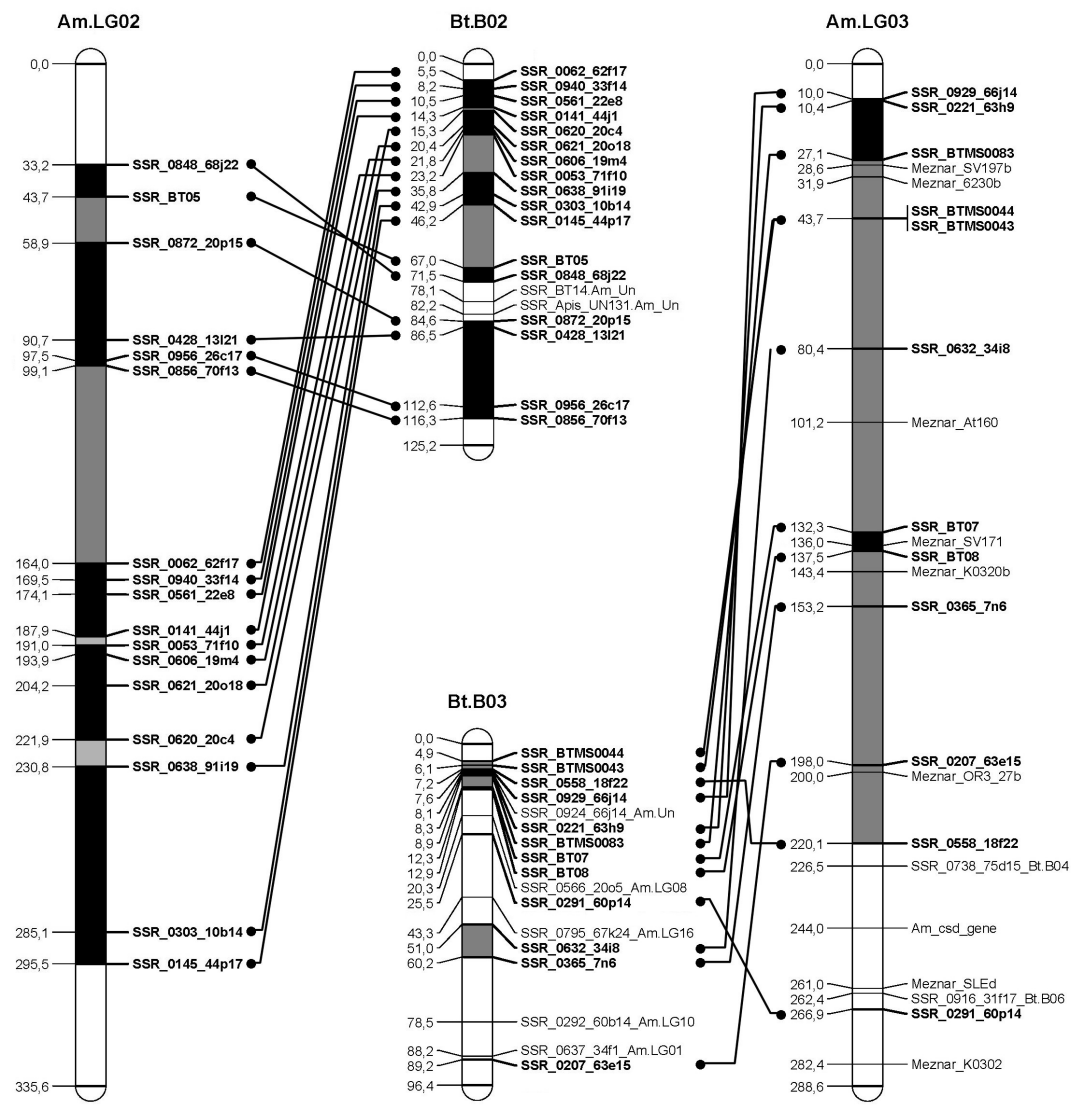
**Comparison between matched *Bombus terrestris *and *Apis mellifera *LGs**. This figure shows the homology between *B. terrestris *and *A. mellifera *LGs 2 and 3. *A. mellifera *LG 3 additionally displays the markers used by ref [85]. Homologous linkage groups of both species are presented next to each other. Bold marker names and connecting lines indicate homologous markers. Black symbolizes synteny; grey indicates intervals between markers, which can be found in the other genome on the same (matched) LG, but rearranged; white intervals are unknown or not syntenic or homologous.

**Figure 3:**
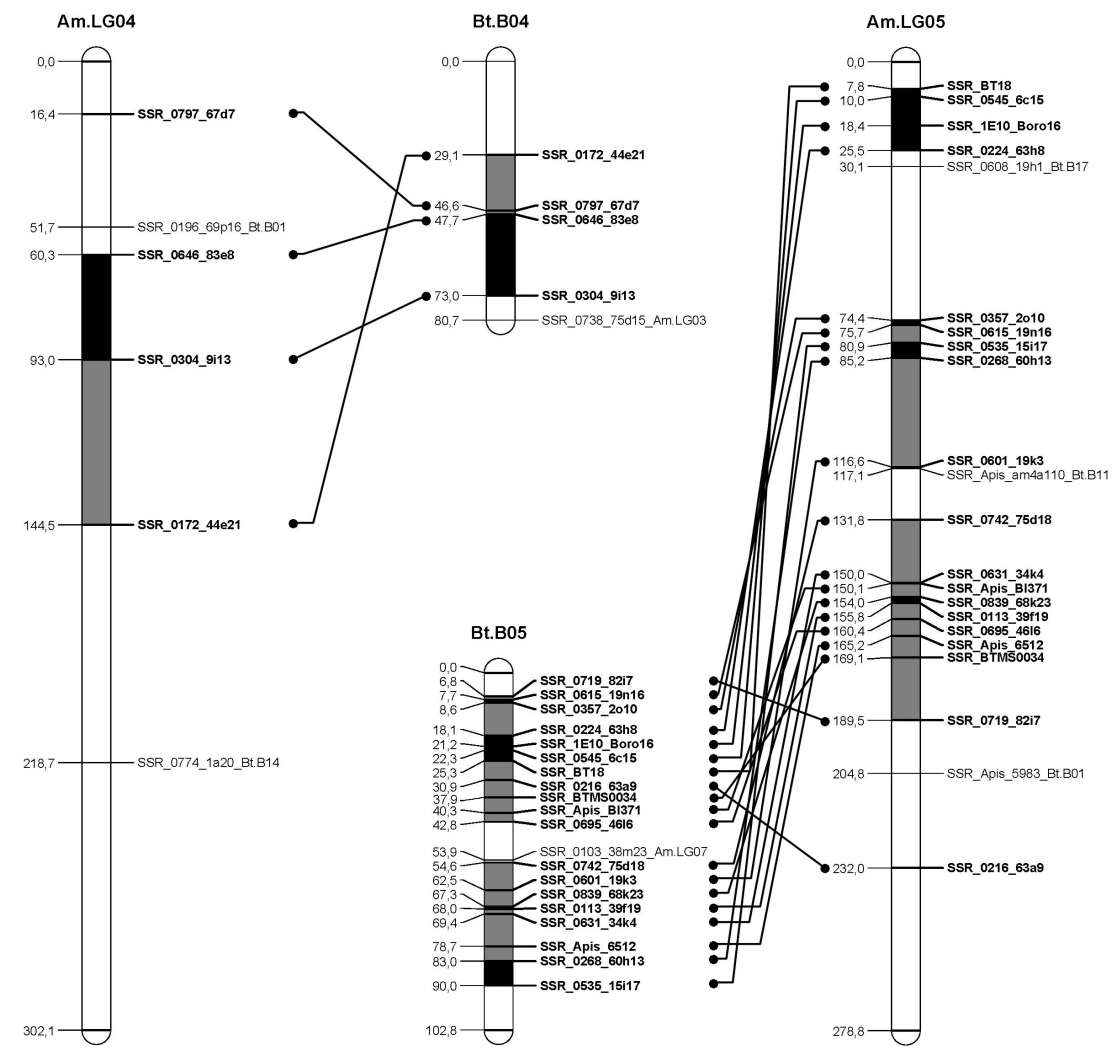
**Comparison between matched *Bombus terrestris *and *Apis mellifera *LGs**. This figure shows the homology between *B. terrestris *and *A. mellifera *LGs 4 and 5. Homologous linkage groups of both species are presented next to each other. Bold marker names and connecting lines indicate homologous markers. Black symbolizes synteny; grey indicates intervals between markers, which can be found in the other genome on the same (matched) LG, but rearranged; white intervals are unknown or not syntenic or homologous.

**Figure 4:**
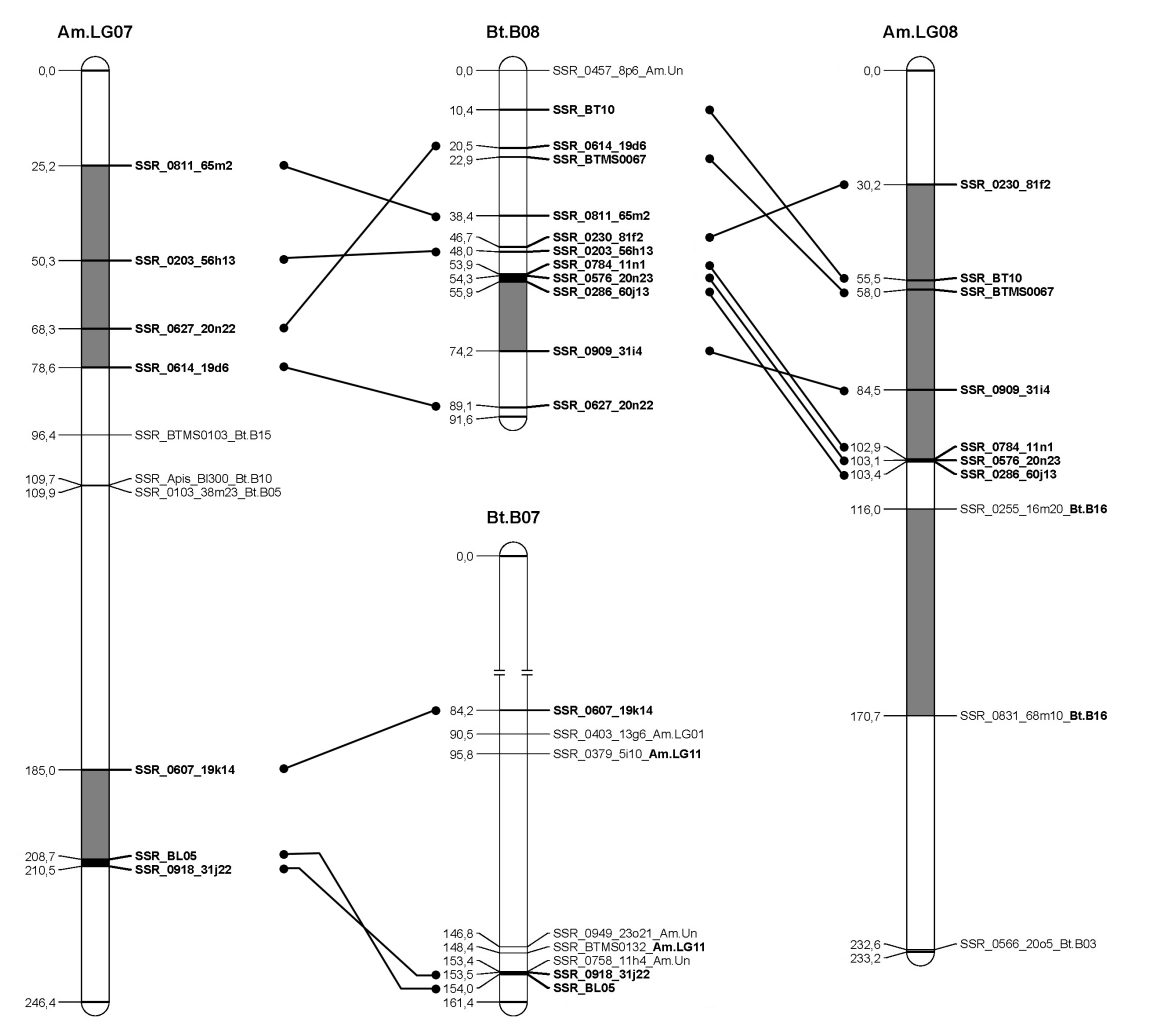
**Comparison between matched *Bombus terrestris *and *Apis mellifera *LGs**. This figure shows the homology between *B. terrestris *and *A. mellifera *LGs 7 and 8. Homologous linkage groups of both species are presented next to each other. Bold marker names and connecting lines indicate homologous markers. Black symbolizes synteny; grey indicates intervals between markers, which can be found in the other genome on the same (matched) LG, but rearranged; white intervals are unknown or not syntenic or homologous.

**Figure 5:**
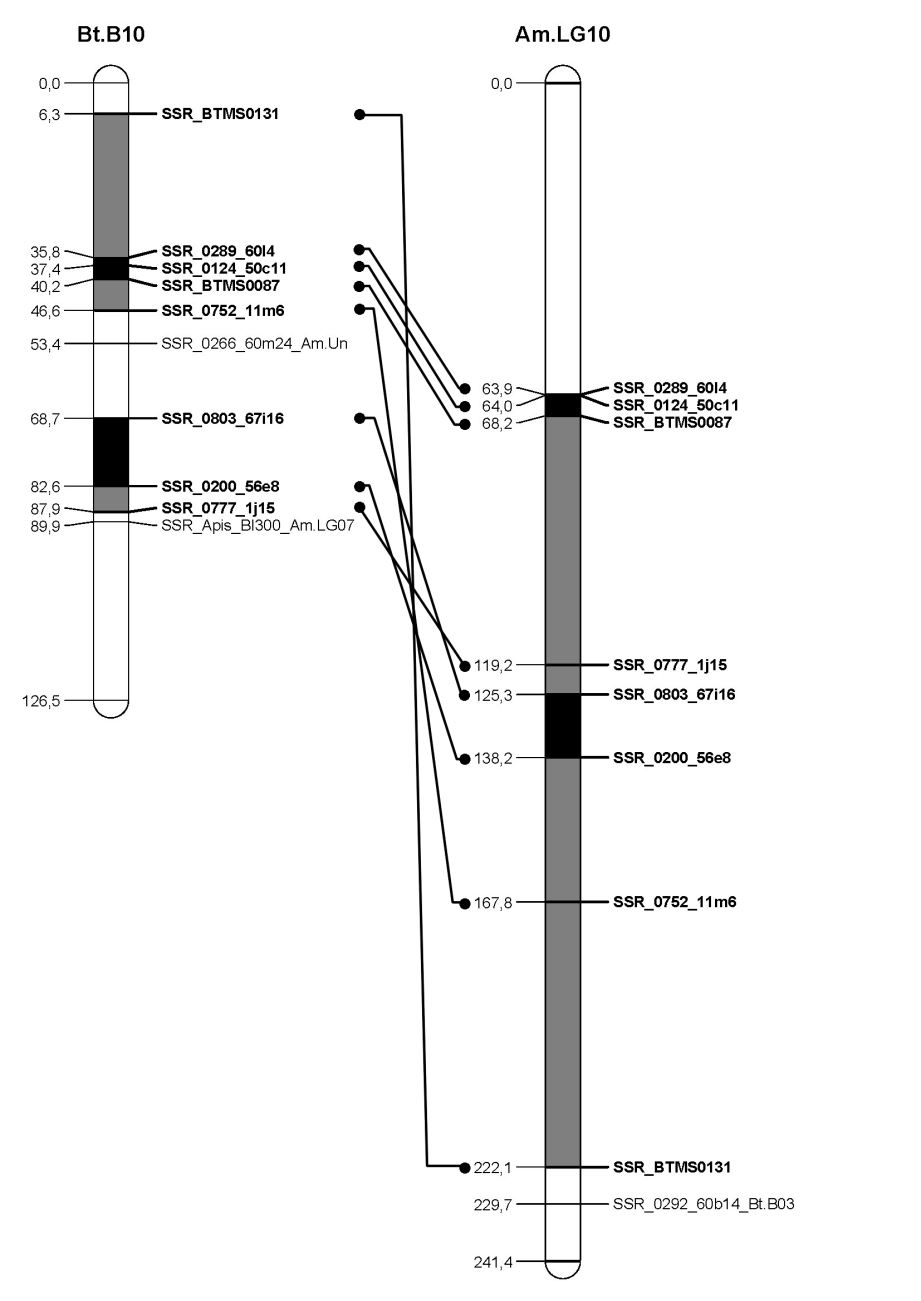
**Comparison between matched *Bombus terrestris *and *Apis mellifera *LGs**. This figure shows the homology between *B. terrestris *and *A. mellifera *LGs 10. Homologous linkage groups of both species are presented next to each other. Bold marker names and connecting lines indicate homologous markers. Black symbolizes synteny; grey indicates intervals between markers, which can be found in the other genome on the same (matched) LG, but rearranged; white intervals are unknown or not syntenic or homologous.

**Figure 6:**
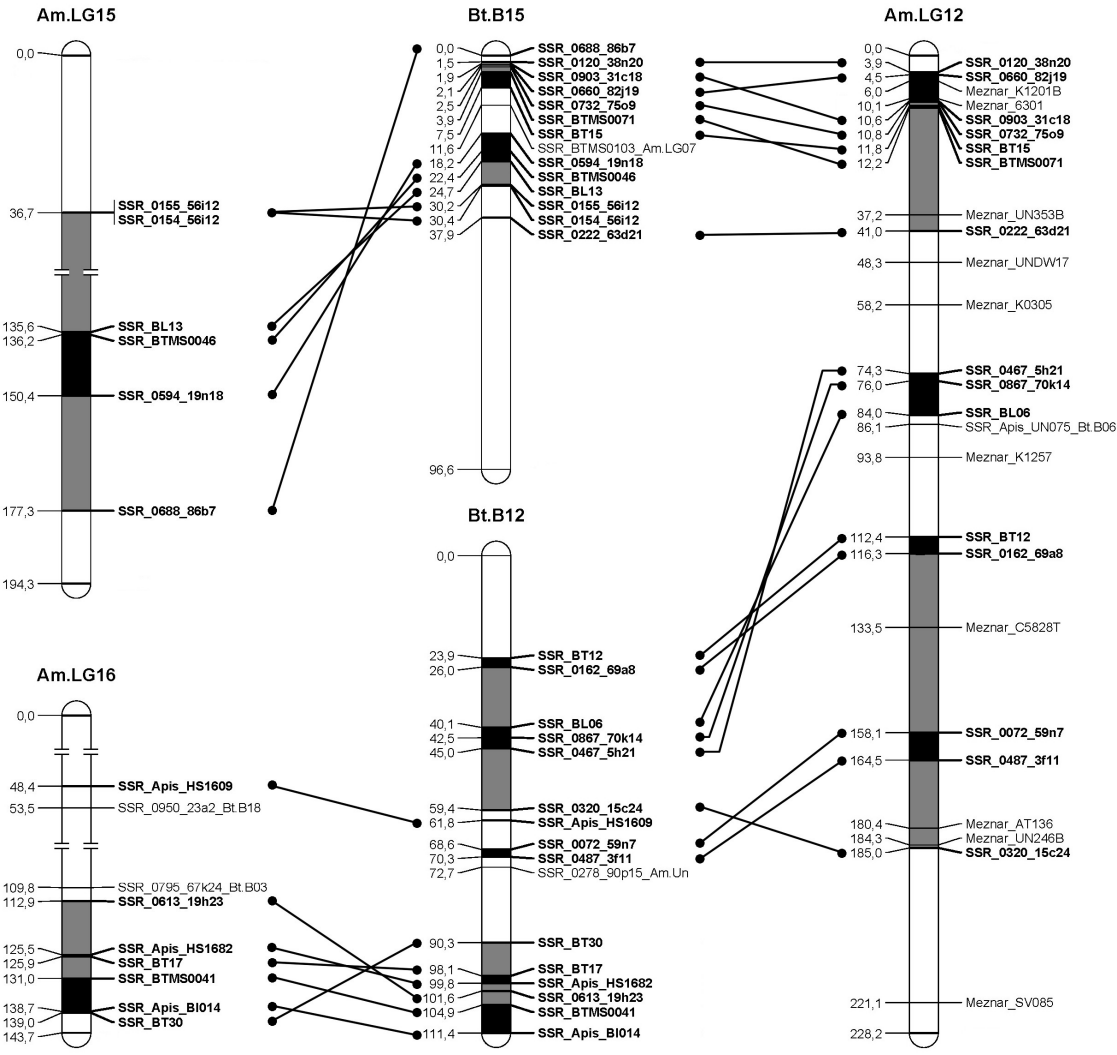
**Comparison between matched *Bombus terrestris *and *Apis mellifera *LGs**. This figure shows the homology between *B. terrestris *12 and 15 and *A. mellifera *LGs 12, 15 and 16. *A. mellifera *LG 12 additionally displays the markers used by ref [85]. Homologous linkage groups of both species are presented next to each other. Bold marker names and connecting lines indicate homologous markers. Black symbolizes synteny; grey indicates intervals between markers, which can be found in the other genome on the same (matched) LG, but rearranged; white intervals are unknown or not syntenic or homologous.

**Figure 7:**
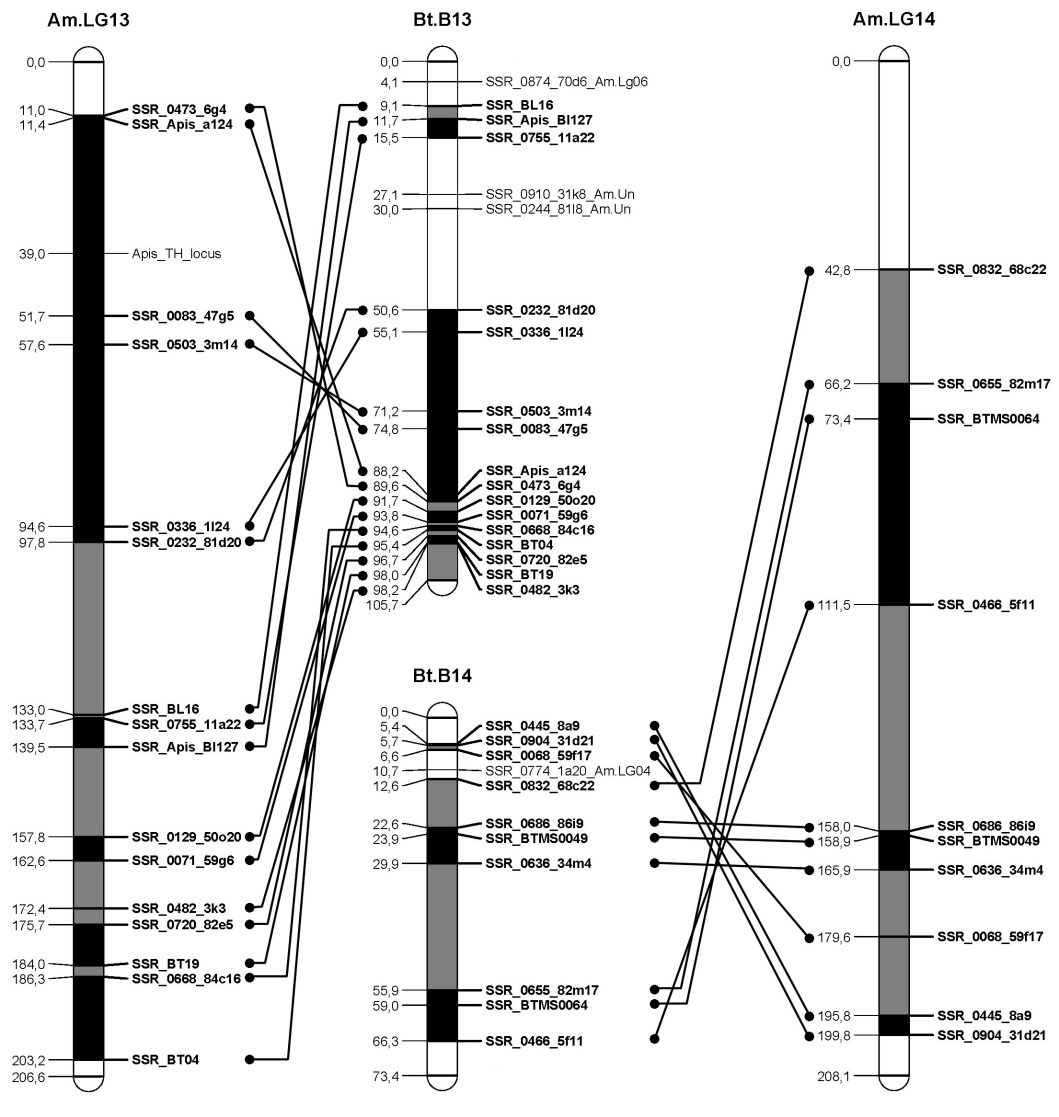
**Comparison between matched *Bombus terrestris *and *Apis mellifera *LGs**. This figure shows the homology between *B. terrestris *and *A. mellifera *LGs 13 and 14. Homologous linkage groups of both species are presented next to each other. Bold marker names and connecting lines indicate homologous markers. Black symbolizes synteny; grey indicates intervals between markers, which can be found in the other genome on the same (matched) LG, but rearranged; white intervals are unknown or not syntenic or homologous.

A *B. terrestris *map containing only the loci homologous to the *A. mellifera *genome was constructed (Figure 1, 2, 3, 4, 5, 6, 7). By comparing both maps, it was possible to homologize 15 of the 18 *B. terrestris *LGs with corresponding *A. mellifera *LGs (Table 2). Omitting homologues to unassigned *A. mellifera *sequences, 10 linkage groups could be precisely matched with 4 to 26 (mean 13.7) homologous loci. In case of LG B02 in *B. terrestris*, all homologous markers match LG 2 in *A. mellifera*. Five *B. terrestris *LGs were composites of parts homologous to two different *A. mellifera *LGs each. 35 homologous loci were mapped on *A. mellifera *LGs that were different from the homologized ones. The three small LGs B16, B17 and B18 consist of too few homologous markers to assign them to *A. mellifera *LGs (Table 2, Additional file 1, Additional file 2).

Overall, there are many conserved chromosomal regions in both genomes. With 83 syntenic marker pairs from 15 bumblebee LGs spanning a total of 302.16 cM in *B. terrestris *and corresponding to 689.80 cM in *A. mellifera*. The distances between syntenic marker pairs ranged from 0.003 to 26.05 cM and from 0.08 to 54.28 cM for *B. terrestris *and *A. mellifera*, respectively (Table 3, Additional file 4). In 18 cases three to six markers were conserved in sequential order. In total the syntenic regions account for 14.81% of the whole map, with the distribution among the different LGs being heterogenic. LGs B02 and B13 show the largest proportion of syntenic regions with 47.99% and 41.66%, respectively. LGs B03 and B07 exhibit the lowest proportion with 1.16% and 0.3% syntenic regions, respectively (Table 3). The mean is 17.6%.

**Table 3:** Summary of the positional information of homologous markers compared between *Bombus terrestris *and *Apis mellifer a*

LG	synteny (n)	**synteny (cM): B.t**.	**synteny (cM): A.m**.	**synteny (cM): ratio B.t./A.m**.	**synteny (%): B.t**.	homology (n)	**homology (cM): B. t**.	**homology (%): B.t**.	inserts (n)	**synteny + homology (%): B.t**.
***Bt.*B01**	12	24.692	57.9	0.43	19.38	10	51.283	40.26	2	59.65

***Bt.*B02**	12	63.332	170.18	0.37	47.99	3	34.315	26.00	0	73.99

***Bt.*B03**	2	1.179	22.03	0.05	1.16	4	15.43	15.21	4	16.38

***Bt.*B04**	1	25.333	32.66	0.78	28.55	2	18.614	20.98	1	49.53

***Bt.*B05**	6	15.837	25.25	0.63	14.44	11	55.54	50.63	1	65.07

***Bt.*B06**	3	8.859	16.37	0.54	4.88	8	55.006	30.30	3	35.19

***Bt.*B07**	1	0.506	1.74	0.29	0.30	0	0	0.00	1	0.30

***Bt.*B08**	2	2.058	0.51	4.04	2.11	1	18.258	18.68	0	20.78

***Bt.*B09**	11	33.858	95.42	0.35	29.11	4	27.785	23.89	4	52.99

***Bt.*B10**	3	18.244	17.15	1.06	13.65	3	41.25	30.86	1	44.50

***Bt.*B11**	6	17.201	26.36	0.65	13.99	2	20.886	16.99	1	30.98

***Bt.*B12**	6	16.823	28.27	0.60	14.26	5	41.498	35.18	0	49.45

***Bt.*B13**	9	46.896	122.42	0.38	41.66	5	7.1	6.31	1	47.97

***Bt.*B14**	5	17.977	57.11	0.31	22.35	3	36.91	45.89	1	68.24

***Bt.*B15**	4	10.327	21.29	0.49	10.08	4	7.885	7.70	1	17.78

***Bt.*B16**	0	0	0	0	0	0	0	0	0	0.00

***Bt.*B17**	0	0	0	0	0	0	0	0	0	0.00

***Bt.*B18**	0	0	0	0	0	0	0	0	0	0.00

**∑/ø**	**∑ 83**	**∑ 303.029**	**∑ 681.56**	**ø 0.45**	**ø 17.59**	**∑ 65**	**∑ 429.815**	**ø 24.59**	**∑ 21**	**ø 42.19**

For each LG information on the number (n) of marker pairs (intervals/segments) is given, which is also present as a marker pair (interval/segment) in *A. mellifera *(synteny). The genetic length in cM of the synthenic intervals/segments in *B. terrestris *(*B.t*.) and the corresponding intervals/segments in *A. mellifera *(*A.m*.) and the ratio between both species is given. Furthermore, the proportion (%) of synthenic marker intervals/segments of the total length of a *B. terrestris *LG is calculated. Likewise numbers of intervals/segments, their length (cM) and proportion (%) of the total length in *B. terrestris *is listed for intervals/segments of markers present in *B. terrestris*, but rearranged (not paired) on the matching *A. mellifera *LG ("homology"), indicating intrachromosomal rearrangements.

*B. terrestris *markers, which were found in a non-matching (see table 2) *A. mellifera *LG (interchromosomal rearrangement) are summed up as "inserts".

The last column shows the proportion (%) of synthenic plus homologous marker intervals/segments for each *B. terrestris *LG.

Most chromosomal regions showed rearrangements in the spatial ordering of markers, but only within the same homologous LG. These cases reflect inversions or non-reciprocal translocations (chromosome mutations) (e.g. Figure 2: B02). While such regions cannot be precisely linked to physical positions on the map as there is no information about the exact locations of breakpoints, these markers are nevertheless located on the same chromosome. A total of 65 such blocks, which do not show an inter-chromosomal insertion, were found in *B. terrestris *and these account for 21.09% (431.76 cM) of the whole map length (Table 3, Additional file 5). The highest proportion of such homologous regions was found on the two LGs B05 and B14 with 50.63% and 45.89%, respectively, whereas the two LGs B07 and B13 with 0 and 6.31%, respectively, showed the lowest proportion: the mean proportion is 24.59% (Table 3).

Taking into account the syntenic and homologous rearranged proportions, a total of 35.9% of the whole map length is conserved between *A. mellifera *and *B. terrestris*. With more than 60% the LGs B02 (73.99%), B14 (68.24%), and B05 (65.07%) exhibit the highest degree of conservation, whereas the lowest degree was observed in LGs B07 (0.3%), B03 (16.38%) and B15 (17.79%) (Table 3): the mean percentage of conservation was 42.19%. Accordingly, a high percentage of the *A. mellifera *LGs are homologous but rearranged if compared to those of *B. terrestris *(Figure 1, 2, 3, 4, 5, 6, 7).

Inter-chromosomal (reciprocal) translocations of larger regions only occurred in five composite chromosomes (see above). Small interchromosomal translocations (a single or double marker insertion) were only observed in 21 cases. Those markers were homologous to *A. mellifera *LGs except for 2, 9, 11, 13, 14 and 15 which had been "inserted" into *B. terrestris *LGs except in LG B02, B08 and B12 (Table 3, Additional file 2).

## Discussion

We here present a second-generation linkage map of the bumblebee *B. terrestris *(Additional file 3). With 18 linkage groups spanning a total of 2'047.09 cM (Table 1) it matches the known number of the haploid chromosomal set (n = 18) [39]. Compared to the previous core linkage map (BBM1, [17]) both the number of LGs (n = 21) and the total map length (2'221.8 cM) are considerably smaller. The shorter map length is a result of a different mapping algorithm compared to that of ref. [17] which used a maximum likelihood algorithm (Mapmaker [48]). The Mapmaker procedure per se assumes no crossover interference causing map inflation whereas the regression algorithm (JoinMap4 [43,49]) used in this study does account for interference hence producing much shorter maps although both algorithms use Kosambi's mapping function [26,49-52]. Since cross-over interference is common in the honeybee and other higher organisms [e.g. [23,53,54]] it seems prudent to consider this mechanism for establishing the bumblebee map. This highlights the importance of choosing a appropriate mapping algorithm to generate comparable and more precise genetic maps. Although several markers showed segregation distortion, those markers were not excluded, since the algorithm (G^2^-statistics for independence) of JoinMap is not affected by segregation distortion [43]. In some case, the Segregation Distortion (meiotic drive) likely is caused by genotype gaps. But it can also have a biological background such as asymmetry of the meiosis (driving allele ends up in the ovocyte instead of in the polar bodies with a probability greater than one half) or can involve gamete destruction (post-meiotic mechanism, e.g. by a selfish segregation distorter genes as found in *Drosophila*, mouse and *Tribolium*). However, our data don't support further assumptions, since the distorted markers are distributed across almost all chromosomes (table 1) without showing a distinct pattern (Additional file 2).

The present 1'902.21 cM map (sizes not corrected for missing chromosome ends) contains 516 markers with an average distance of 4.02 cM between markers. By reanalyzing the original data set used to create the core linkage map [17], we found that the map size was increased only by 16.6% (271.21 cM) by including 277 additional markers (map sizes not corrected for missing chromosome ends). The genome coverage (92.92%) is much higher than the previous map's 81% [17]. 99.99% of the genome is located within a distance of 17.6 cM to a marker. The current map is thus nearly saturated and thus a valuable tool for further QTL mapping studies [2,3].

The two prior estimates for physical genome size were both based on flow cytometry and muscle cells, but differed substantially. Ref. [16] estimated a genome size of 274 Mb whereas ref. [17] reported an estimate of 625 Mb. The staining method used by [16] is typically biased towards the AT content of the genome [55-58] and hence may have lead to underestimating the genome size of *B. terrestris *because the 61% AT content is only 6.3% less than that of *A. mellifera *[46]. By correcting for the AT bias the *B. terrestris *genome size is estimated to be 433 Mb, very similar to the estimate of 426.41 Mb in this study derived from the relation of the measured genetic and known physical distance between two neighboring markers. Theses concurring measurements lead us to the conclusion that the genome size reported by ref. [17] was overestimated. There is a further estimate of about 250 Mb based on preliminary data for the *B. terrestris *genome assembly (Baylor College of Medicine Human Genome Sequencing Center, unpublished), but this need to be verified.

Given a physical genome size of 430 Mb, the estimated recombination density of 4.76 cM/Mb for *B. terrestris *is slightly higher than the 4.42 cM/Mb previously published [17]. Although this recombination rate is much less than that of the honeybee genome (15.7 cM/Mb [46]) it is still a high value compared to other eukaryotic organisms (Vertebrata 1.37 cM/Mb, Insecta excl. Hymenoptera 2.69 cM/Mb, [46,59]). This supports the idea that a high genomic recombination rate may be positively correlated with other genomic traits such as AT content, as shown for several organism groups with the exception of mammals [46]. Alternatively, a high recombination rate might have evolved due to sex-restricted recombination (e.g. haplo-diploid Hymenoptera) or may be related to sociality in insects as such [59]. Social Hymenoptera show a higher recombination rate (mean 10.27 cM/Mb, n = 4) than non-social parasitoid Hymenoptera (mean 3.99 cM/Mb, n = 4) [46,59]. Depending on the *B. terrestris *genome size in the final genome assembly, the recombination rate in the bumblebee might be significantly higher than estimated here. Based on a preliminary value of 250 Mb a very high genome wide recombination rate of 8.19 cM/Mb is calculated making the relationships discussed above even more clear. However, the sample size for data on genomes from different taxonomic groups is still low, therefore a robust conclusion is not yet possible.

Using sequence similarities, it was possible to unambiguously match 15 linkage groups between *B. terrestris *and *A. mellifera, *of which five were composites consisting of partial homologous to two *A. mellifera *LGs (Table 2). A high proportion (21%) of the genome showed homology in terms of markers present on the homologous LG, and 14.81% were identified as synteny blocks, segments with preserved marker order without disruption by rearrangements [60,61]. The genomic homology is most striking at the level of individual LGs. More than 40% of LG B02 and B13 are syntenic. If synteny and rearranged blocks are added, on average a total of 42.19% of a LG is conserved. Three LGs even show a conservation of more than 65% (Table 3).

This homology and synteny can be used to refer to previously mapped quantitative trait loci (QTLs) or genes in the honeybee (as shown above, Additional file 1). These loci may now serve as target candidate regions for the same traits in the bumblebee; hence, the map we present here can be a valuable tool for cross-species genetic mapping. For example the thelytoky locus of *A. mellifera *[62] is located on chromosome nr. 13, at 39 cM between the syntenic marker pair SSR_Apis_a124 (11.4 cM) and SSR_0083_47g5 (51.66 cM) (Figure 7, Additional file 2, 4). In *B. terrestris *this pair is located on LG B13 (88.2 cM and 74.8 cM, respectively). It is thus conceivable that the corresponding gene is located between the same markers in *B. terrestris*.

A biologically important element is the sex locus (*csd *gene [63]), which is located on *A. mellifera *chromosome nr. 3, at 243.95 cM. In the present new map there is unfortunately no syntenic marker pair surrounding this locus. The neighboring homologous markers are located on *Bombus *LG B04 and B06, whereas the remaining part of the chromosome is mostly homologous to LG B03 (Figure 1, 2, 3, Additional file 2, 4, 5). While this locus has already been mapped directly in *B. terrestris *too [16], it cannot be homologized with the honeybee, as the sex locus was linked only to RAPD markers. Hence, there is no unambiguous information for the location of the corresponding sex locus *csd *in *B. terrestris*. Its identification may require information on the whole genome sequence of the bumblebee [64-66].

Comparisons of genome architecture can provide insights into genome and chromosome evolution [65,67,68]. As we have shown, there is a high degree of homology between the genomes of *B. terrestris *and *A. mellifera*. On the other hand the divergence time between the bumblebees (tribe Bombini) and the honeybees (tribe Apini) has been roughly estimated based on fossil records and several phylogenetic or molecular systematic studies [69-74]. From this data, Bombini and Meliponini are considered to be sister groups, with the split of the Bombini (plus the Meliponini) and the Apini to have occurred 125 - 80 million years ago (mya) (mean ~ 100 mya), coinciding with the Angiosperm radiation [75,76]. The genera *Bombus *and *Apis *are considered to have radiated much later into today's species diversity [71,72]. Despite an independent evolution of about 100 million years, large parts of the genome and even almost entire chromosomes are relatively conserved.

Other comparative genomic studies have revealed various degrees of conservation between genomes of species with different divergence times. In the genus *Drosophila *(age ~ 40 mya), for example extensive gene shuffling within the homologous chromosome arms between even moderately diverged genomes such as *D. melanogaster *and *D. erecta *(divergence ~ 10 mya [77]) is observed. The conservation of the genetic architecture between *D. melanogaster *and *Rhagoletis pomonella *(Diptera, Tephritidae) (divergence ~ 50-55 mya) was high in chromosomes X and 3, respectively, whereas *D. melanogaster *chromosome 2 is composed of regions homologous to all five *R. pomonella *LGs with many inter-chromosomal rearrangements [37]. In mammalian genomes, extensive shuffling of chromosomal regions between species (e.g. human, elephant, horse, hedgehog, cattle, cat, mouse) of phylogenetically different lineages, which split about 90 mya has been reported too [36,64,78-83]. Even within short evolutionary times (<40 mya) extensive genome reorganizations have been reported among the anthropoid Primates [84]. These exceed the differences between bumblebees and honeybees by far, although insects usually have much shorter generation lengths. Clearly many more rearrangements, both intra- and inter-chromosomal, have occurred among genomes of taxa with a similar divergence time as between *B. terrestris *and *A. mellifera*.

In light of these other studies, the large degree of homology between *B. terrestris *and *A. mellifera *is rather surprising. In fact, similar levels of homology as observed here are typical for very closely related species, such as mouse and rat (divergence 16 mya [64]) or with the example of the conserved marker order in chromosomes 3 and 12 of *Apis mellifera *and *A. florea *[85], which split 20-25 mya [71]. The high level of homology is furthermore surprising in light of the high genome-wide recombination rate of both bee species, which clearly exceed the average recombination rate in insects or vertebrates [46,59].

Our findings suggest a very slow rate of genome and chromosomal evolution in these two bee species. This supports the previous conclusions that the honeybee genome evolved more slowly than that of the fruitfly or *Anopheles *mosquitoes [20]. Our new data and the conservation of marker order between two *Apis *species [85], suggests that the genome and chromosome evolution might be slow in the whole family Apinae.

Reasons for such a slow evolutionary rate at the genome level remain elusive. The relative lack of retrotransposons in *A. mellifera *[20] or the high density of simple-sequence-repeats (SSR, microsatellites) might be important factors. Sociality, which occurs in all four Apinae tribes, or haplodiploidy could also favor a slow genome evolution or vice versa. With the advance of next generation sequencing, it will clearly be only a matter of time until the whole genome sequence of *Bombus terrestris *and other bee species will be available. This will then allow us to conduct a comprehensive genomic comparison to unravel the ultimate evolutionary causes of the high genome conservation in social bees.

## Conclusions

This report describes the construction of the first saturated linkage map for *Bombus terrestris *with 516 mapped markers. The genome coverage is ~93%. Based on homologies of microsatellite flanking sequences to the genome of *Apis mellifera *it was possible to match 15 linkage groups. A genome comparison revealed that about 15% of the genome is organized in syntenic blocks and 21% in rearranged regions on the same homologized linkage group. Inter-chromosomal rearrangements are less frequent. This high conservation of the genetic architecture is unexpected since both bee species exhibit a very high recombination rate and a long divergence time. This map will be an essential tool for QTL mapping, with the high degree of homology potentially allowing for cross species mapping in *B. terrestris *and *A. mellifera*.

## Methods

### Mapping population & DNA extraction

A *B. terrestris *colony (BBM-1) was established as a phase-known mapping population with 577 male individuals [17]. It originated from a mated and hibernated queen from a wild catch in northwestern Switzerland. We used the same specimens (males) from this colony for this mapping study as well. DNA from the bumblebee individuals was extracted using the DNeasy Blood & Tissue Kit (QIAGEN) following the manual.

### Genetic markers, PCR, genotyping

End sequencing of a BAC-library [19] was carried out according to [86] and a screen for 1-5 bp simple sequence repeats (SSRs, microsatellites) was done using MISA [87]. Complete sequences containing the SSRs were checked against each other and already existing SSRs [12-15] for redundancy, employing a local BLAST search in BioEdit [88] or using the MAFFT alignment algorithm [89]. Primer pairs were designed with BatchPrimer3 [90], Primer3Plus [91] or manually for the resulting unique SSR loci. PCR was carried out at 50°C, 55°C and 60°C using a TGradient thermocycler (Biometra) to optimize reaction conditions. Standard PCR reactions were performed in a total volume of 15 μL (~10 ng DNA, 0.25 μL of each primer (10 μM), 2.25 μL of 10x reaction buffer (160 mM (NH_4_)_2_SO_4_, 670 mM Tris-HCl, 15 mM MgCl_2_, 0.1% Tween 20), 0.13 μM of a mix of each dNTP (10 mM) and 0.3 U *Taq *polymerase (GeneCraft), 3 min at 94°C, 37 cycles of 45 s at 94°C, 45 s at 50-60°C and 45 s at 72°C, 3 min at 72°C). The PCR products were visualized on a 2% agarose gel stained with ethidium bromide and successfully amplifying loci were then checked for polymorphism in *B. terrestris *by performing a standard PCR containing a DNA pool from 11 *B. terrestris *queens or females (species identity was confirmed according to [92]) from Estonia (Tartu), France (Arles, Normandy), Hungary (Debrecen), Ireland (Belfast), Sweden (Tovetorp), Belgium (Zemst), Norway (Kalvøya), Austria (Vienna), laboratory colony (Koppert) and Germany (Halle) (5 ng each). The PCR products were run on a QIAxcel automatic capillary electrophoresis (QIAxcel DNA High Resolution Kit) and analyzed using the QIAxcel BioCalculator software (QIAGEN).

For a subset of polymorphic loci as well as the 123 microsatellite loci for *B. terrestris *recently published by ref. [15] fluorescent labeled primers (FAM, HEX or TET, Metabion) were used in multiplex standard PCR reactions (containing three primer pairs with a different fluorescent label and 20 ng of a DNA pool from 10 males) to detect informative (dimorphic) loci in the mapping population. The PCR products were run on a MegaBace capillary sequencer and analyzed using the FragmentProfiler software. Additional loci were tested with unlabelled primer pairs in single locus PCR containing also 20 ng of a DNA pool from 10 males of the mapping population and PCR products were run and analyzed on the QIAxcel system (see above).

The genotyping of 288 to 384 males from the mapping population was performed in multiplex PCRs with 2 - 10 primer pairs depending on fragment size and fluorescent label. Multiplex PCRs with fluorescent labeled primer pairs were conducted using PCR Master Mix (Promega) and then run and analyzed on the MegaBace system (see above). Multiplex PCR's with unlabeled primer pairs were conducted using the standard PCR procedure (see above) and were run on the QIAxcel system (see above).

Worker-produced males were already detected and excluded by [17]. However, two more individuals with paternal alleles were detected and excluded from further analysis.

Preliminary information for an additional estimate of the genome size was obtained from Baylor College of Medicine Human Genome Sequencing Center (http://www.hgsc.bcm.tmc.edu).

### Genotype analysis & map construction

For analysis of the genotypes the software JoinMap 4.0 [43] was used. The segregation was tested against the normal Mendelian expectation ration using a Chi^2 ^test in order to detect Segregation Distortion. The software first detects linkage groups (LGs) based on the independence LOD (larger than 5) calculated for the recombination frequencies and the linkage phase is automatically determined using pairs with a LOD larger than 5. The mapping was done phase-unknown using marker pair LOD scores of 5 or higher. Ref. [17] confirmed that in this system prior knowledge of linkage phase is not necessary for accurate genetic mapping (no difference between phase-known and phase-unknown mapping). Furthermore, the phase for some loci on each LG is known from [17], so the correct phase of each marker could be established. Then, for each LG, marker order and genetic distance were inferred by regression mapping using Kosambi's mapping function [50] to account for crossover interference. Three rounds were performed, using linkages with a recombination frequency smaller than 0.40 and a LOD larger than 1.0. After adding a single locus a "ripple" (test for all possible 3-point orders of consecutive markers to obtain the most likely order for every marker) was performed using linkage information from up to 10 neighboring markers to verify that the marker order found in previous analyses was correct. Maps were printed with the MapChart 2.2 software [93].

### Homology of SSR loci

Using the available sequencing information for each mapped SSR (whole clone sequence containing the microsatellite, 337 to 961 bp) we performed a cross-species MegaBlast or alternatively BlastN search against the *Apis mellifera *genome (NCBI, Amel 4.0). Unique Blast hits with a homologous sequence larger than 30 bp, a score higher than 45 or a maximal identity of higher than 67% were used (two exceptions were made, where one of the characteristics had fallen below one of the thresholds). By plotting the genetic map [23] onto the physical map [94,20], the genetic position on the respective *A. mellifera *linkage group could be estimated from the physical sequence homology (Blast hit). Next, individual maps for each linkage group of *B. terrestris *were plotted, only containing the homologous markers. Similarly all *A. mellifera *LGs were plotted again only using the homologous markers from the *A. mellifera *map. Both genomes were then compared side by side in MapChart2.2 [93].

## Authors' contributions

ES carried out most of the experimental and conceptional work, microsatellite development, primer design and testing, genotyping and data analysis, map construction and drafting the manuscript. LW, RS and PS carried out the testing and genotyping of the *A. mellifera *primers, screened BAC sequences for microsatellites and provided original data and the mapping population from [17]. RR and MK carried out the BAC library end sequencing. RFAM was responsible for project conception, contributed drafting the manuscript and participated together with PS, RS and LW in design and coordination. All authors read and approved the final manuscript.

## Supplementary Material

Additional file 1 **SSR markers and Blast results**. This table lists all used microsatellite markers. For novel SSR markers the GenBank accession numbers, the primer sequences with their annealing temperatures (Ta), the repeat motif, the SSR type (c - composite, p - pure, number indicating the motif length), an approximate size range of the PCR fragment, an approximate number of alleles (N_a_), the BAC_ID (source of the repeat sequence) and (if applied) a fluorescent label are given. For all SSR markers the location (LG) in *Bombus terrestris *and *Apis mellifera *map, the origin/source is listed and the results of the Blast search against the *Apis mellifera *genome (Amel_4.0) as well as the Blast method are presented. Click here for file

Additional file 2 **Mapping data**. For each mapped marker (AFLP and SSR) the genetic position on the LG, the distance (interval) to the next (following) marker and the genetic position within the *A. mellifera *genome (if a homologue was found) is given. Furthermore the linkage phase in the used mapping population is given. Significance (p-value) of segregation distortion (Chi^2 ^test of allele frequencies for deviation from Mendelian segregation ratio) is indicated by stars (*:0.1; **:0.05; ***:0.01; ****:0.005; *****:0.001; ******:0.0005; *******:0.0001). Click here for file

Additional file 3 ***Bombus terrestris *linkage map**. This plot shows the *Bombus terrestris *linkage map with absolute marker positions and marker names for each linkage group. Click here for file

Additional file 4 **Synteny**. This table shows syntenic marker pairs (intervals/segments) with their location (LG) and interval/segment length (cM) in *B. terrestris *(*B.t.*) and the corresponding interval/segment in *A. mellifera *(*A.m*.), as well as their ratio. Click here for file

Additional file 5 **Homology**. This table shows marker pairs (intervals/segments) from the *B. terrestris *(*B.t.*) map, of which both markers are located on a matching (see table 2) *A. mellifera *(*A.m*.) LG, but rearranged (not paired, hence no synteny). Their LG (*B.t*.) and interval/segment length (cM, *B.t*.) is given. Click here for file

## References

[B1] Velthuis HHW van Doorn A A century of advances in bumblebee domestication and the economic and environmental aspects of its commercialization for pollination Apidologie 2006 37 4 421 451 10.1051/apido:2006019

[B2] Wilfert L Gadau J Baer B Schmid-Hempel P Natural variation in the genetic architecture of a host-parasite interaction in the bumblebee *Bombus terrestris* Molecular Ecology 2007 16 6 1327 1339 10.1111/j.1365-294X.2007.03234.x 17391417

[B3] Wilfert L Gadau J Schmid-Hempel P The genetic architecture of immune defense and reproduction in male *Bombus terrestris *bumblebees Evolution 2007 61 4 804 815 10.1111/j.1558-5646.2007.00079.x 17439613

[B4] Baer B Schmid-Hempel P Unexpected consequences of polyandry for parasitism and fitness in the bumblebee, *Bombus terrestris* Evolution 2001 55 8 1639 1643 11580023 10.1111/j.0014-3820.2001.tb00683.x

[B5] Baer B Bumblebees as model organisms to study male sexual selection in social insects Ecology and Sociobiology 2003 54 6 521 533 10.1007/s00265-003-0673-5

[B6] Pereboom JJM Jordan WC Sumner S Hammond RL Bourke AFG Differential gene expression in queen-worker caste determination in bumble-bees Proceedings of the Royal Society B-Biological Sciences 2005 272 1568 1145 1152 10.1098/rspb.2005.3060 PMC1559810 16024376

[B7] Kraus RB Wolf S Moritz RFA The Male flight distance and population substructure in the bumblebee *Bombus terrestris* Journal of Animal Ecology 2009 78 1 247 252 10.1111/j.1365-2656.2008.01479.x 19120605

[B8] Plowright RC Laverty TM The ecology and sociobiology of the bumblebees Review of Entomology 1984 29 175 199 10.1146/annurev.en.29.010184.001135

[B9] Leadbeater E Chittka L The dynamics of social learning in an insect model, the bumblebee (*Bombus terrestris*) Behavioral Ecology and Sociobiology 2007 61 11 1789 1796 10.1007/s00265-007-0412-4

[B10] Goulson D Bumblebees - their behavior and Ecology 2003 New York: Oxford University Press

[B11] Schluns H Sadd BM Schmid-Hempel P Crozier RH Infection with the trypanosome *Crithidia bombi *and expression of immune-related genes in the bumblebee *Bombus terrestris* Developmental and Comparative Immunology 2010 34 7 705 709 10.1016/j.dci.2010.02.002 20144650

[B12] Estoup A Solignac M Harry M Cornuet JM Characterization of (GT)_n _and (CT)_n _microsatellites in 2 insect species - *Apis mellifera *and *Bombus terrestris.* Nucleic Acids Research 1993 21 6 1427 1431 10.1093/nar/21.6.1427 8464734 PMC309328

[B13] Estoup A Tailliez C Cornuet JM Solignac M Size homoplasy and mutational processes of interrupted microsatellites in 2 bee species, *Apis mellifera *and *Bombus terrestris *(Apidae) Molecular Biology and Evolution 1995 12 6 1074 1084 8524041 10.1093/oxfordjournals.molbev.a040282

[B14] Reber-Funk C Schmid-Hempel R Schmid-Hempel P Microsatellite loci for *Bombus *spp Molecular Ecology Notes 2006 6 1 83 86 10.1111/j.1471-8286.2005.01147.x

[B15] Stolle E Rohde M Vautrin D Solignac M Schmid-Hempel P Schmid-Hempel R Moritz RFA Novel microsatellite DNA loci for *Bombus terrestris *(Linnaeus, 1758) Molecular Ecology Resources 2009 9 5 1345 1352 10.1111/j.1755-0998.2009.02610.x 21564905

[B16] Gadau J Gerloff CU Kruger N Chan H Schmid-Hempel P Wille A Page RE A linkage analysis of sex determination in *Bombus terrestris *(L.) (Hymenoptera : Apidae) Heredity 2001 87 234 242 10.1046/j.1365-2540.2001.00919.x 11703515

[B17] Wilfert L Gadau J Schmid-Hempel P A core linkage map of the bumblebee *Bombus terrestris* Genome 2006 49 10 1215 1226 10.1139/G06-075 17213903

[B18] Sadd BM Kube M Klages S Reinhardt R Schmid-Hempel P Analysis of a normalised expressed sequence tag (EST) library from a key pollinator, the bumblebee *Bombus terrestris* BMC Genomics 2010 11 10.1186/1471-2164-11-110 20156341 PMC2838840

[B19] Wilfert L Torres MM Reber-Funk C Schmid-Hempel R Tomkins J Gadau J Schmid-Hempel P Construction and characterization of a BAC-library for a key pollinator, the bumblebee *Bombus terrestris *L Insectes Sociaux 2009 56 1 44 48 10.1007/s00040-008-1034-1

[B20] Weinstock GM Robinson GE Gibbs RA Worley KC Evans JD Maleszka R Robertson HM Weaver DB Beye M Bork P Insights into social insects from the genome of the honeybee *Apis mellifera* Nature 2006 443 7114 931 949 10.1038/nature05260 17073008 PMC2048586

[B21] Erickson DL Fenster CB Stenoien HK Price D Quantitative trait locus analyses and the study of evolutionary process Molecular Ecology 2004 13 9 2505 2522 10.1111/j.1365-294X.2004.02254.x 15315666

[B22] Wu R Ma CX Casella G Statistical Genetics of Quantitative Traits - Linkage, Maps, and QTL 2007 Berlin, Heidelberg: Springer

[B23] Solignac M Mougel F Vautrin D Monnerot M Cornuet JM A third-generation microsatellite-based linkage map of the honey bee, Apis mellifera, and its comparison with the sequence-based physical map Genome Biology 2007 8 10.1186/gb-2007-8-3-403 PMC1896015 17459148

[B24] Brondani RPV Williams ER Brondani C Grattapaglia D A microsatellite-based consensus linkage map for species of Eucalyptus and a novel set of 230 microsatellite markers for the genus Bmc Plant Biology 2006 6 10.1186/1471-2229-6-20 PMC1599733 16995939

[B25] Criscione CD Valentim CLL Hirai H LoVerde PT Anderson TJC Genomic linkage map of the human blood fluke Schistosoma mansoni Genome Biology 2009 10 6 10.1186/gb-2009-10-6-r71 19566921 PMC2718505

[B26] Hong YB Chen XP Liang XQ Liu HY Zhou GY Li SX Wen SJ Holbrook CC Guo BZ A SSR-based composite genetic linkage map for the cultivated peanut (Arachis hypogaea L.) genome Bmc Plant Biology 2010 10 10.1186/1471-2229-10-17 PMC2835713 20105299

[B27] Itoh T Watanabe T Ihara N Mariani P Beattie CW Sugimoto Y Takasuga A A comprehensive radiation hybrid map of the bovine genome comprising 5593 loci Genomics 2005 85 4 413 424 10.1016/j.ygeno.2004.12.007 15780744

[B28] Kucuktas H Wang SL Li P He CB Xu P Sha ZX Liu H Jiang YL Baoprasertkul P Somridhivej B Construction of Genetic Linkage Maps and Comparative Genome Analysis of Catfish Using Gene-Associated Markers Genetics 2009 181 4 1649 1660 10.1534/genetics.108.098855 19171943 PMC2666527

[B29] Lorenzen MD Doyungan Z Savard J Snow K Crumly LR Shippy TD Stuart JJ Brown SJ Beeman RW Genetic linkage maps of the red hour beetle, Tribolium castaneum, based on bacterial artificial chromosomes and expressed sequence tags Genetics 2005 170 2 741 747 10.1534/genetics.104.032227 15834150 PMC1450394

[B30] McKay SD Schnabel RD Murdoch BM Aerts J Gill CA Gao C Li C Matukumalli LK Stothard P Wang Z Construction of bovine whole-genome radiation hybrid and linkage maps using high-throughput genotyping Animal Genetics 2007 38 2 120 125 10.1111/j.1365-2052.2006.01564.x 17302794 PMC2063635

[B31] Miles LG Isberg SR Glenn TC Lance SL Dalzell P Thomson PC Moran C A genetic linkage map for the saltwater crocodile (Crocodylus porosus) Bmc Genomics 2009 10 10.1186/1471-2164-10-339 19640266 PMC2907706

[B32] Raudsepp T Gustafson-Seabury A Durkin K Wagner ML Goh G Seabury CM Brinkmeyer-Langford C Lee EJ Agarwala R Stallknecht-Rice E A 4,103 marker integrated physical and comparative map of the horse genome Cytogenetic and Genome Research 2008 122 1 28 36 10.1159/000151313 18931483 PMC2587302

[B33] Remington DL Whetten RW Liu BH O'Malley DM Construction of an AFLP genetic map with nearly complete genome coverage in Pinus taeda Theoretical and Applied Genetics 1999 98 8 1279 1292 10.1007/s001220051194 12238515

[B34] Sanetra M Henning F Fukamachi S Meyer A A Microsatellite-Based Genetic Linkage Map of the Cichlid Fish, Astatotilapia burtoni (Teleostei): A Comparison of Genomic Architectures Among Rapidly Speciating Cichlids Genetics 2009 182 1 387 397 10.1534/genetics.108.089367 18757932 PMC2674835

[B35] Wang S Zhang LL Meyer E Matz MV Construction of a high-resolution genetic linkage map and comparative genome analysis for the reef-building coral Acropora millepora Genome Biology 2009 10 11 10.1186/gb-2009-10-11-r126 19900279 PMC3091320

[B36] Murphy WJ Larkin DM Everts-van der Wind A Bourque G Tesler G Auvil L Beever JE Chowdhary BP Galibert F Gatzke L Dynamics of mammalian chromosome evolution inferred from multispecies comparative maps Science 2005 309 5734 613 617 10.1126/science.1111387 16040707

[B37] Roethele JB Romero-Severson J Feder JL Evidence for broad-scale conservation of linkage map relationships between Rhagoletis pomonella (Diptera : Tephritidae) and Drosophila melanogaster (Diptera : Drosophilidae) Annals of the Entomological Society of America 2001 94 6 936 947 10.1603/0013-8746(2001)094[0936:EFBSCO]2.0.CO;2

[B38] Wilfert L Schmid-Hempel P Gadau J Hunter W, Kole C Bumblebee, *Bombus terrestris* Genome Mapping and Genomics in Animals 2008 1 Berlin, Heidelberg: Springer Verlag 17 25 full_text

[B39] Hoshiba H Duchateau MJ Velthuis HHW Diploid Males in the Bumblebee *Bombus terrestris *(Hymenoptera), Karyotype Analyses of Diploid Females, Diploid Males and Haploid Males Japanese Journal of Entomology 1995 63 1 203 207

[B40] Haberl M Tautz D Tri- and tetranucleotide microsatellite loci in honey bees (*Apis mellifera*) - a step towards quantitative genotyping Molecular Ecology 1999 8 8 1358 1360 10.1046/j.1365-294X.1999.00701_5.x 10507874

[B41] Solignac M Vautrin D Baudry E Mougel F Loiseau A Cornuet JM A microsatellite-based linkage map of the Honeybee, *Apis mellifera *L Genetics 2004 167 1 253 262 10.1534/genetics.167.1.253 15166152 PMC1470837

[B42] Solignac M Vautrin D Loiseau A Mougel F Baudry E Estoup A Garnery L Haberl M Cornuet JM Five hundred and fifty microsatellite markers for the study of the honeybee (*Apis mellifera *L.) genome Molecular Ecology Notes 2003 3 2 307 311 10.1046/j.1471-8286.2003.00436.x

[B43] Van Oojen JW Kyazma B.V JoinMap 4, Software for the calculation of genetic linkage maps in experimental populations 2006 Wageningen

[B44] Fishman L Kelly AJ Morgan E Willis JH A genetic map in the *Mimulus **guttatus *species complex reveals transmission ratio distortion due to heterospecific interactions Genetics 2001 159 4 1701 1716 11779808 10.1093/genetics/159.4.1701 PMC1461909

[B45] Lange K Boehnke M How many polymorphic genes will it take to span the human genome American Journal of Human Genetics 1982 34 6 842 845 6960692 PMC1685701

[B46] Lattorff HMG Moritz RFA Recombination rate and AT-content show opposite correlations in mammalian and other animal genomes Evolutionary Biology 2008 35 2 146 149 10.1007/s11692-008-9019-6

[B47] Gregory TR Nicol JA Tamm H Kullman B Kullman K Leitch IJ Murray BG Kapraun DF Greilhuber J Bennett MD Eukaryotic genome size databases Nucleic Acids Research 2007 35 D332 D338 10.1093/nar/gkl828 17090588 PMC1669731

[B48] Lander ES Green P Abrahamson J Barlow A Daly MJ Lincoln SE Newburg L Mapmaker - an interactive computer package for constructing primary genetic linkage maps of experimental and natural populations Genomics 1987 1 2 174 181 10.1016/0888-7543(87)90010-3 3692487

[B49] Stam P Construction of integrated genetic-linkage maps by means of a new computer package - Joinmap Plant Journal 1993 3 5 739 744 10.1111/j.1365-313X.1993.00739.x

[B50] Kosambi DD The estimation of map distances from recombination values Ann Eugenic 1943 12 172 175

[B51] Qi XQ Stam P Lindhout P Comparison and integration of four barley genetic maps Genome 1996 39 2 379 394 10.1139/g96-049 18469901

[B52] Liebhard R Koller B Gianfranceschi L Gessler C Creating a saturated reference map for the apple (*Malus *× *domestica *Borkh.) genome Theoretical and Applied Genetics 2003 106 8 1497 1508 12677403 10.1007/s00122-003-1209-0

[B53] Mancera E Bourgon R Brozzi A Huber W Steinmetz LM High-resolution mapping of meiotic crossovers and non-crossovers in yeast Nature 2008 454 7203 479 U471 10.1038/nature07135 18615017 PMC2780006

[B54] Zhao HY Speed TP McPeek MS Statistical-analysis of crossover interference using the chi-square model Genetics 1995 139 2 1045 1056 7713407 10.1093/genetics/139.2.1045 PMC1206355

[B55] Button DK Robertson BR Determination of DNA content of aquatic bacteria by flow cytometry Applied and Environmental Microbiology 2001 67 4 1636 1645 10.1128/AEM.67.4.1636-1645.2001 11282616 PMC92780

[B56] Dolezel J Greilhuber J Plant genome size estimation by flow cytometry: Inter-laboratory comparison Annals of Botany 1998 82 17 26 10.1006/anbo.1998.0730

[B57] Johnston JS Bennett MD Reference standards for determination of DNA content of plant nuclei American Journal of Botany 1999 86 5 609 613 10.2307/2656569 10330063

[B58] Bosco G Campbell P Analysis of drosophila species genome size and satellite DNA content reveals significant differences among strains as well as between species Genetics 2007 177 3 1277 1290 10.1534/genetics.107.075069 18039867 PMC2147996

[B59] Wilfert L Gadau J Schmid-Hempel P Variation in genomic recombination rates among animal taxa and the case of social insects Heredity 2007 98 4 189 197 10.1038/sj.hdy.6800950 17389895

[B60] Nadeau JH Taylor BA Lengths of chromosomal segments conserved since divergence of man and mouse Proceedings of the National Academy of Sciences of the United States of America-Biological Sciences 1984 81 3 814 818 10.1073/pnas.81.3.814 PMC344928 6583681

[B61] Pevzner P Tesler G Genome Rearrangements in mammalian evolution: Lessons from human and mouse genomes Genome Research 2003 13 1 37 45 10.1101/gr.757503 12529304 PMC430962

[B62] Lattorff HMG Moritz RFA Control of reproductive dominance by the *thelytoky *gene in honeybees Biology Letters 2007 3 3 292 295 10.1098/rsbl.2007.0083 17412668 PMC2464700

[B63] Beye M Hasselmann M The gene *csd *is the primary signal for sexual development in the honeybee and encodes an SR-type protein Cell 2003 114 4 419 429 10.1016/S0092-8674(03)00606-8 12941271

[B64] Bourque G Pevzner PA Tesler G Reconstructing the genomic architecture of ancestral mammals: Lessons from human, mouse, and rat genomes Genome Research 2004 14 4 507 516 10.1101/gr.1975204 15059991 PMC383294

[B65] Faraut T Addressing chromosome evolution in the whole-genome sequence era Chromosome Research 2008 16 1 5 16 10.1007/s10577-007-1208-0 18293102

[B66] Stark A Lin MF Kheradpour P Pedersen JS Parts L Carlson JW Crosby MA Rasmussen MD Roy S Deoras AN Discovery of functional elements in 12 *Drosophila *genomes using evolutionary signatures Nature 2007 450 219 232 10.1038/nature06340 17994088 PMC2474711

[B67] Hoshiba H Chromosome Evolution of Bees and Wasps (Hymenoptera, Apocrita) on the Basis of C-banding Pattern Analyses Japanese Journal of Entomology 1993 61 3 465 492

[B68] Rascol VL Pontarotti P Levasseur A Ancestral animal genomes reconstruction Current Opinion in Immunology 2007 19 542 546 10.1016/j.coi.2007.06.009 17702562

[B69] Cameron SA Mardulyn P Multiple molecular data sets suggest independent origins of highly eusocial behavior in bees (Hymenoptera: Apinae) Systematic Biology 2001 50 2 194 214 10.1080/10635150151125851 12116928

[B70] Danforth BN Sipes S Fang J Brady SG The history of early bee diversification based on five genes plus morphology Proceedings of the National Academy of Sciences of the United States of America 2006 103 41 15118 15123 10.1073/pnas.0604033103 17015826 PMC1586180

[B71] Engel MS A giant honey bee from the Middle Miocene of Japan (Hymenoptera : Apidae) American Museum Novitates 2006 3504 1 12

[B72] Hines HM Historical biogeography, divergence times, and diversification patterns of bumblebees (Hymenoptera : Apidae : *Bombus*) Systematic Biology 2008 57 1 58 75 10.1080/10635150801898912 18275002

[B73] Michener CD Grimaldi DA The oldest fossil bee - Apoid history, evolutionary stasis, and antiquity of social-behavior Proceedings of the National Academy of Sciences of the United States of America 1988 85 17 6424 6426 10.1073/pnas.85.17.6424 16593976 PMC281984

[B74] Whitfield JB Kjer KM Ancient rapid radiations of insects: Challenges for phylogenetic analysis Annual Review of Entomology 2008 53 449 472 10.1146/annurev.ento.53.103106.093304 17877448

[B75] Lidgard S Crane PR Quantitative analyses of the early angiosperm radiation Nature 1988 331 6154 344 346 10.1038/331344a0

[B76] Wing SL Evolution and Expansion of flowering plants Paleontological Society Papers 2000 6 209 231

[B77] Clark AG Eisen MB Smith DR Bergman CM Oliver B Markow TA Kaufman TC Kellis M Gelbart W Iyer VN Evolution of genes and genomes on the *Drosophila *phylogeny Nature 2007 450 7167 203 218 10.1038/nature06341 17994087

[B78] Chowdhary BP Raudsepp T Fronicke L Scherthan H Emerging patterns of comparative genome organization in some mammalian species as revealed by Zoo-FISH Genome Research 1998 8 6 577 589 9647633 10.1101/gr.8.6.577

[B79] Everts van der Wind A Larkin DM Green CA Elliott JS Olmstead CA Chiu R Schein JE Marra MA Womack JE Lewin HA A high-resolution whole-genome cattle-human comparative map reveals details of mammalian chromosome evolution Proceedings of the National Academy of Sciences of the United States of America 2005 102 51 18526 18531 10.1073/pnas.0509285102 16339895 PMC1317968

[B80] Frönicke L Wienberg J Stone G Adams L Stanyon R Towards the delineation of the ancestral eutherian genome organization: comparative genome maps of human and the African elephant (*Loxodonta africana*) generated by chromosome painting Proceedings of the Royal Society of London Series B-Biological Sciences 2003 270 1522 1331 1340 10.1098/rspb.2003.2383 PMC1691379 12965023

[B81] Meyers SN Rogatcheva MB Larkin DM Yerle M Milan D Hawken RJ Schook LB Beever JE Piggy-BACing the human genome - II. A high-resolution, physically anchored, comparative map of the porcine autosomes Genomics 2005 86 6 739 10.1016/j.ygeno.2005.04.010 16246521

[B82] Milinkovitch MC Helaers R Depiereux E Tzika AC Gabaldon T 2x genomes - depth does matter Genome Biology 2010 11 2 10.1186/gb-2010-11-2-r16 20144222 PMC2872876

[B83] Yang FT Graphodatsky AS Li TL Fu BY Dobigny G Wang JH Perelman PL Serdukova NA Su WT O'Brien PCM Comparative genome maps of the pangolin, hedgehog, sloth, anteater and human revealed by cross-species chromosome painting: further insight into the ancestral karyotype and genome evolution of eutherian mammals Chromosome Research 2006 14 3 283 296 10.1007/s10577-006-1045-6 16628499

[B84] Stanyon R Rocchi M Capozzi O Roberto R Misceo D Ventura M Cardone MF Bigoni F Archidiacono N Primate chromosome evolution: Ancestral karyotypes, marker order and neocentromeres Chromosome Research 2008 16 1 17 39 10.1007/s10577-007-1209-z 18293103

[B85] Meznar ER Gadau J Koeniger N Rueppell O Comparative linkage mapping suggests a high recombination rate in all honeybees J Hered 2010 101 Suppl 1 S118 126 10.1093/jhered/esq002 20212006

[B86] Kuhl H Beck A Wozniak G Canario AV Volckaert FA Reinhardt R The European sea bass *Dicentrarchus labrax *genome puzzle: comparative BAC-mapping and low coverage shotgun sequencing BMC Genomics 2010 11 1 68 10.1186/1471-2164-11-68 20105308 PMC2837037

[B87] MISA- MIcroSAtellite identification tool [pgrcipk-gaterslebende/misa/]

[B88] Hall TA BioEdit: a user-friendly biological sequence alignment editor and analysis program for Windows 95/98/NT Nucleic Acids Symposium Series 1999 41 95 98

[B89] Katoh K Kuma K Toh H Miyata T MAFFT version 5: improvement in accuracy of multiple sequence alignment Nucleic Acids Research 2005 33 2 511 518 10.1093/nar/gki198 15661851 PMC548345

[B90] You FM Huo NX Gu YQ Luo MC Ma YQ Hane D Lazo GR Dvorak J Anderson OD BatchPrimer3: A high throughput web application for PCR and sequencing primer design BMC Bioinformatics 2008 9 10.1186/1471-2105-9-253 18510760 PMC2438325

[B91] Untergasser A Nijveen H Rao X Bisseling T Geurts R Leunissen JAM Primer3Plus, an enhanced web interface to Primer3 Nucleic Acids Research 2007 35 W71 W74 10.1093/nar/gkm306 17485472 PMC1933133

[B92] Murray TE Fitzpatrick U Brown MJF Paxton RJ Cryptic species diversity in a widespread bumblebee complex revealed using mitochondrial DNA RFLPs Conservation Genetics 2008 9 3 653 666 10.1007/s10592-007-9394-z

[B93] Voorrips RE MapChart: Software for the graphical presentation of linkage maps and QTLs Journal of Heredity 2002 93 1 77 78 10.1093/jhered/93.1.77 12011185

[B94] Chakravarti A A graphical representation of genetic and physical maps - the Marey map Genomics 1991 11 1 219 222 10.1016/0888-7543(91)90123-V 1765381

