# Supplementary figures and images for: A second generation genetic map of the bumblebee Bombus terrestris (Linnaeus, 1758) reveals slow genome and chromosome evolution in the Apidae
Source: BMC Genomics. 2011 Jan 19;12:48. doi: 10.1186/1471-2164-12-48 (PMC3034698; doi:10.1186/1471-2164-12-48)

## B01

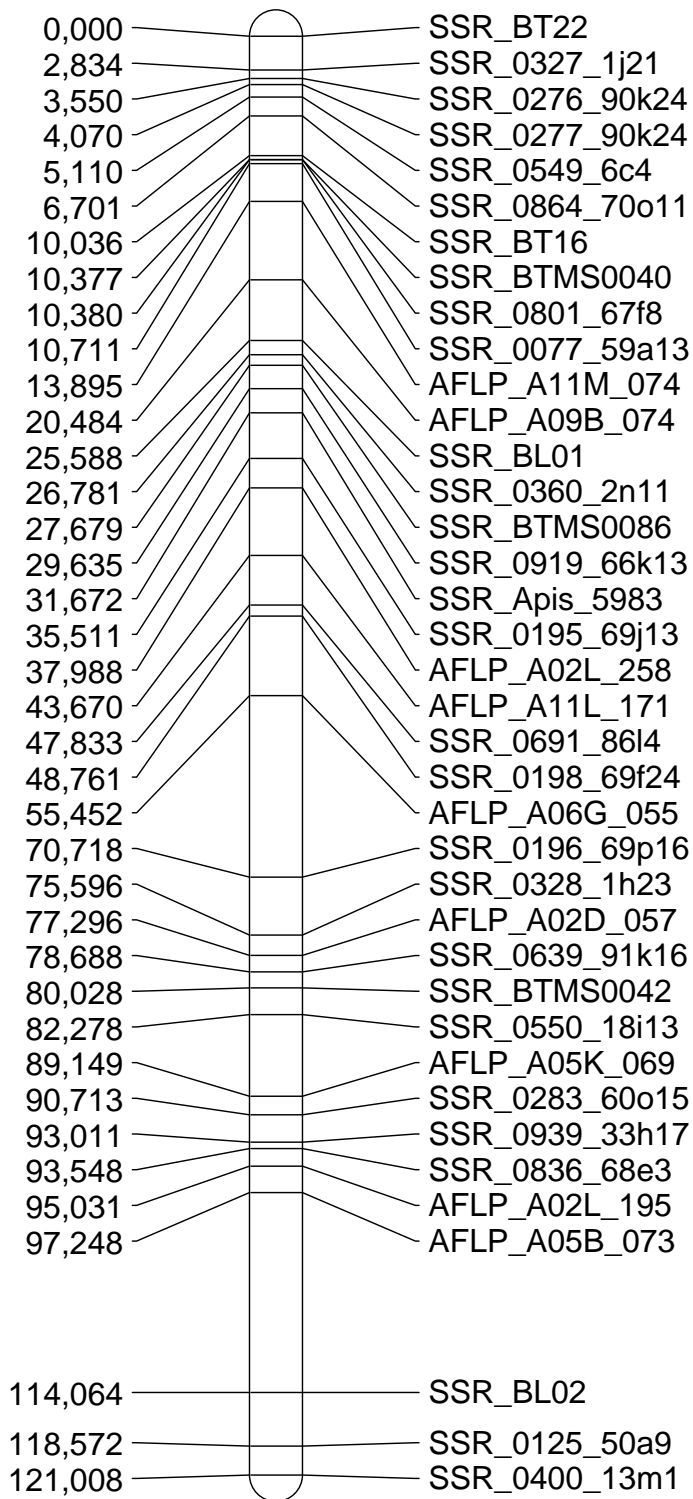

## B02

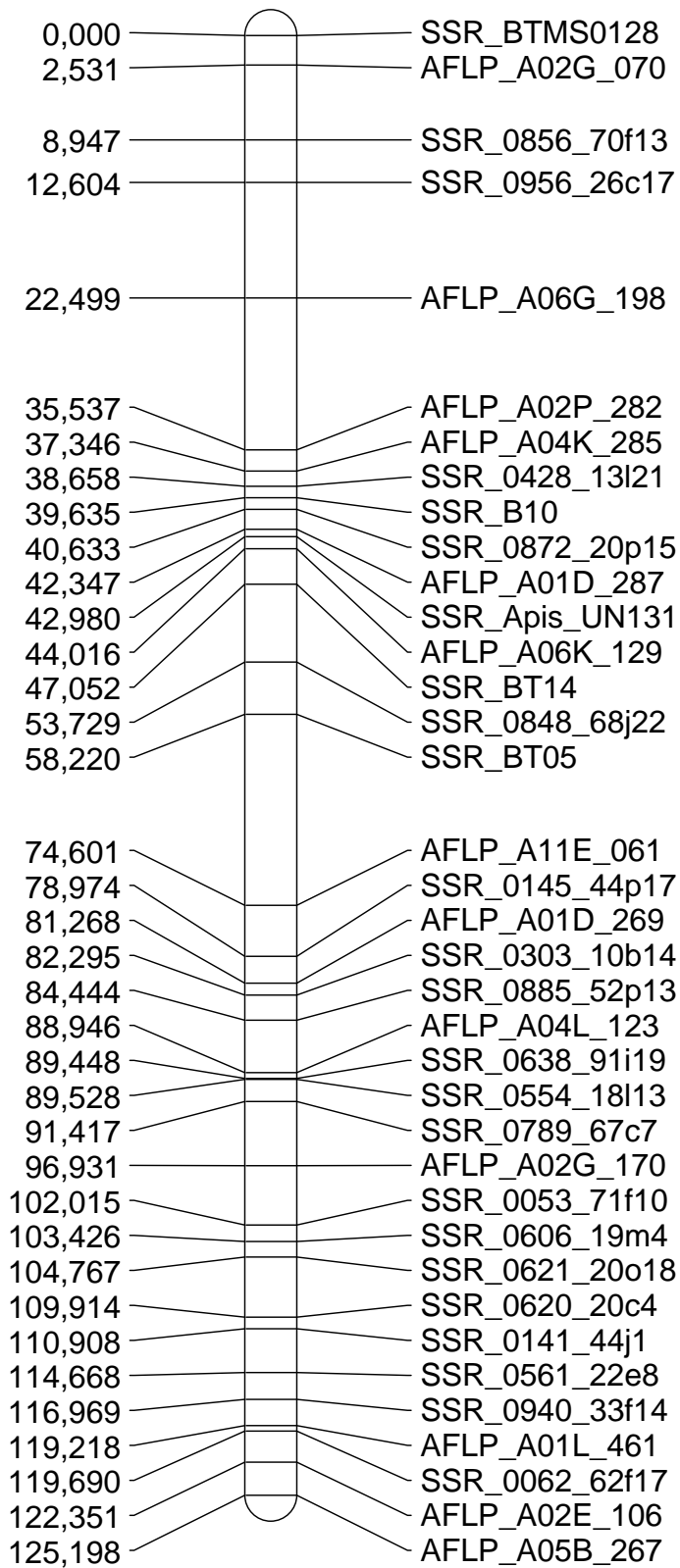

## B03

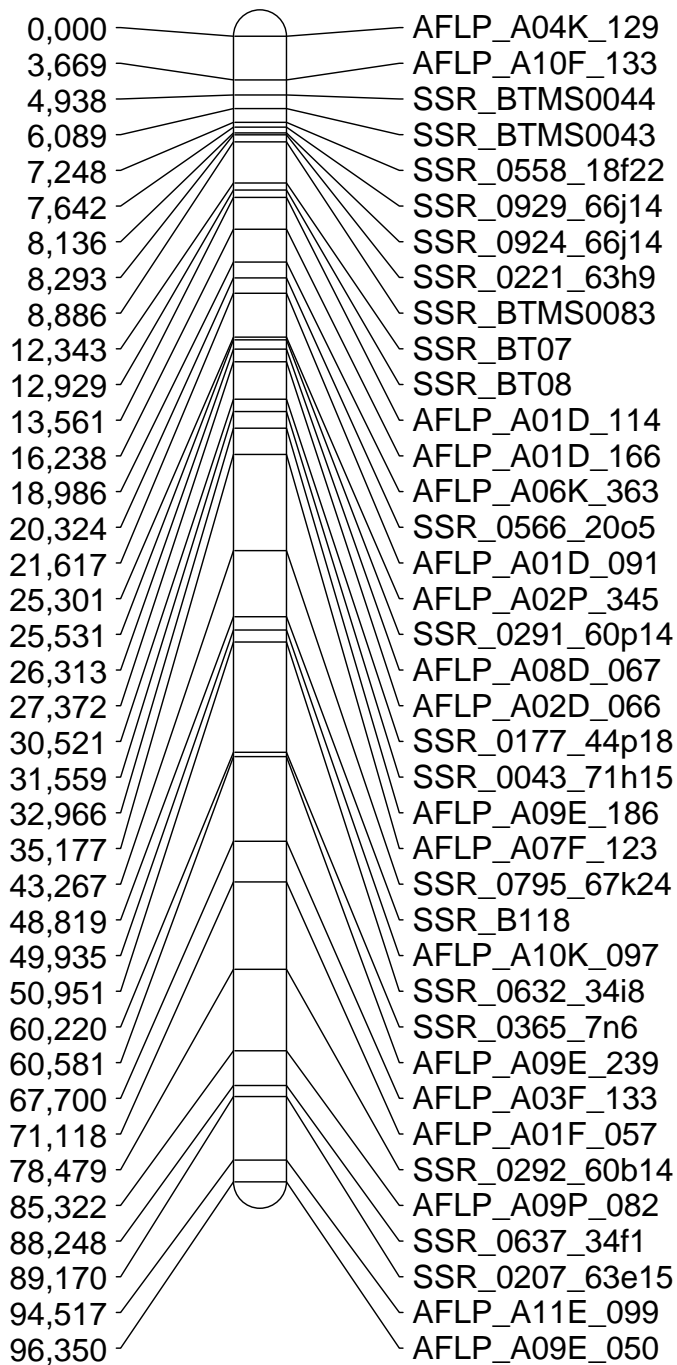

# B04

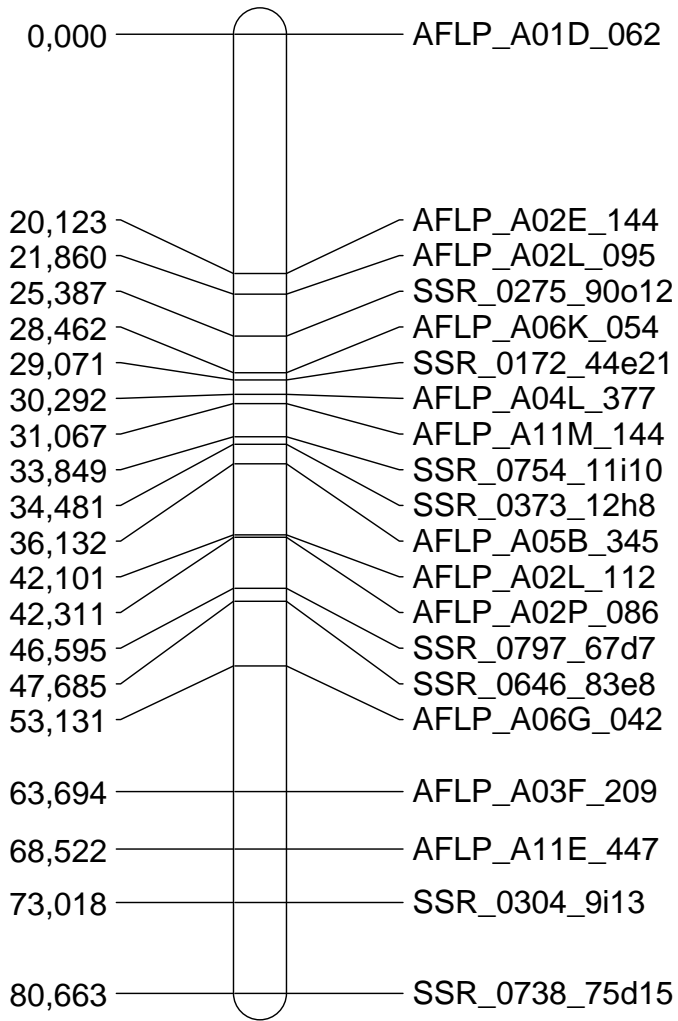

# B05

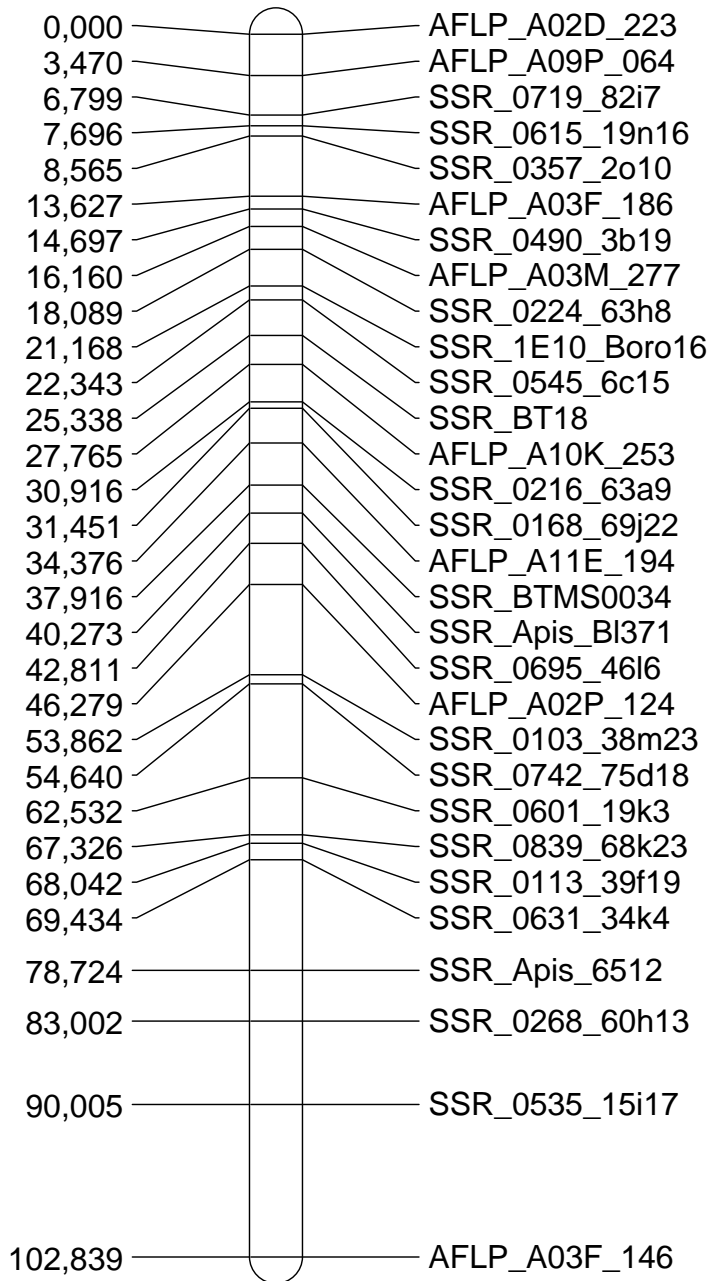

# B06

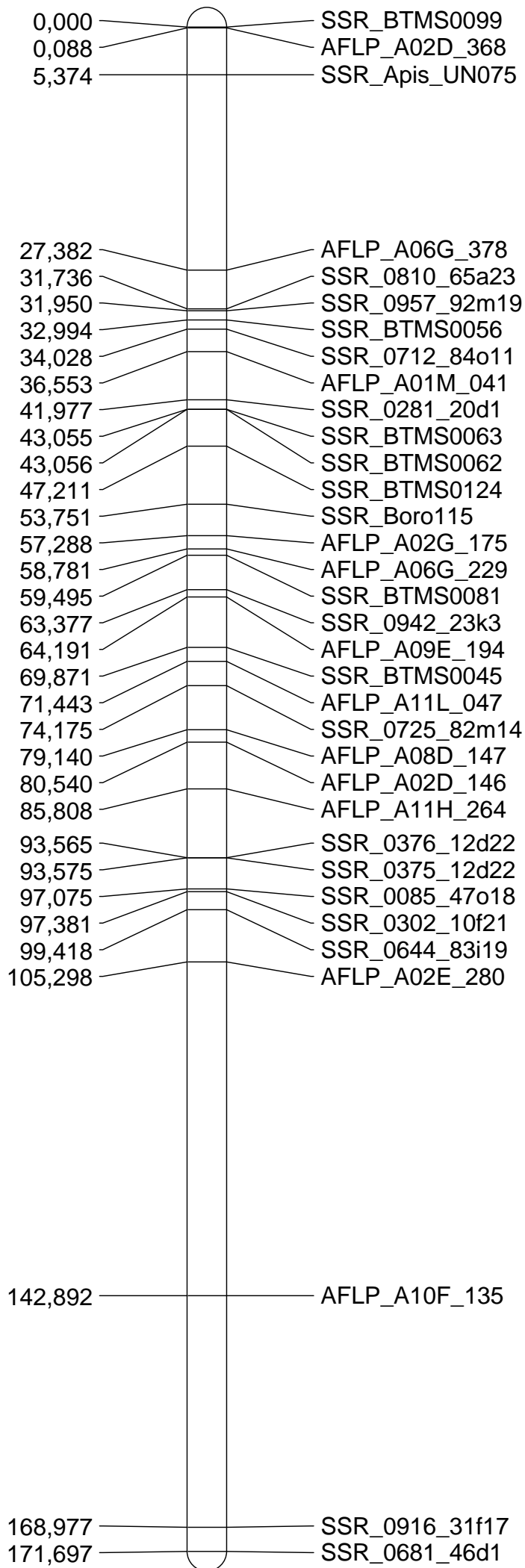

# B07

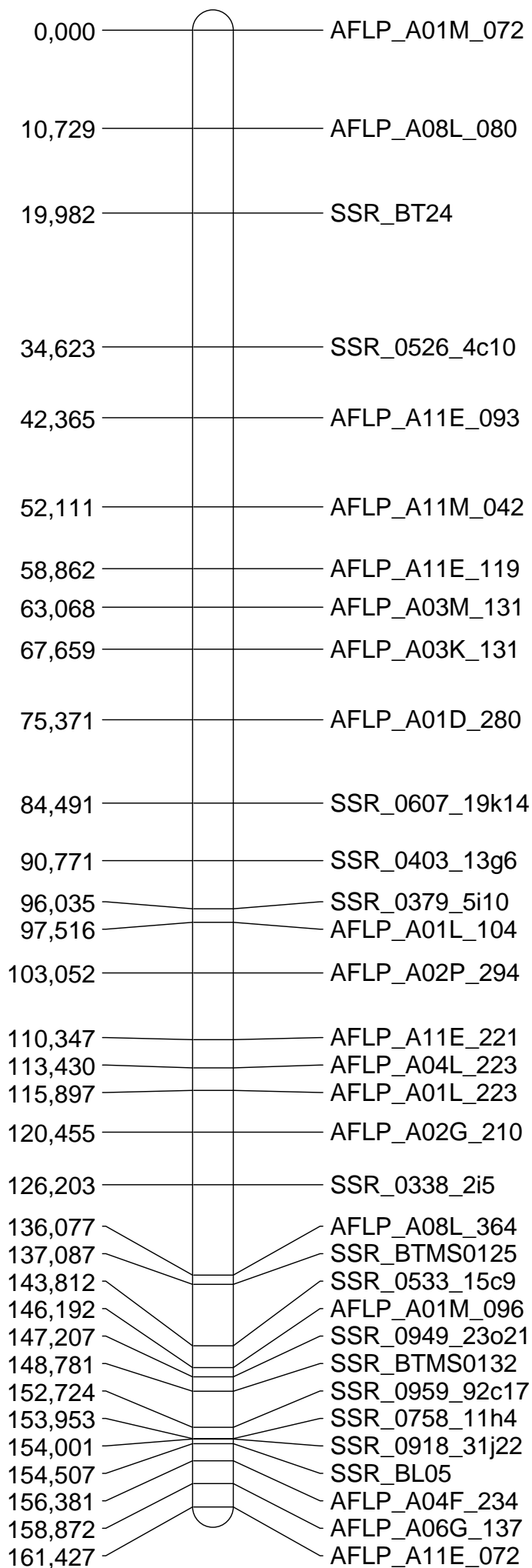

# B08

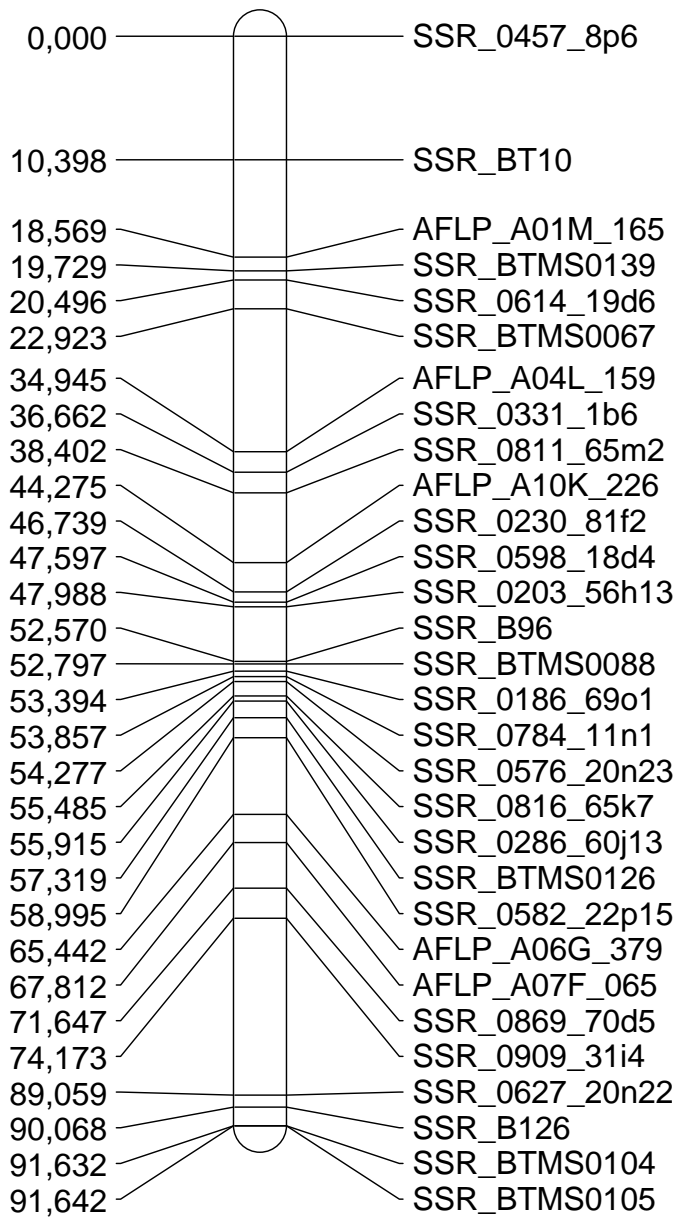

## B09

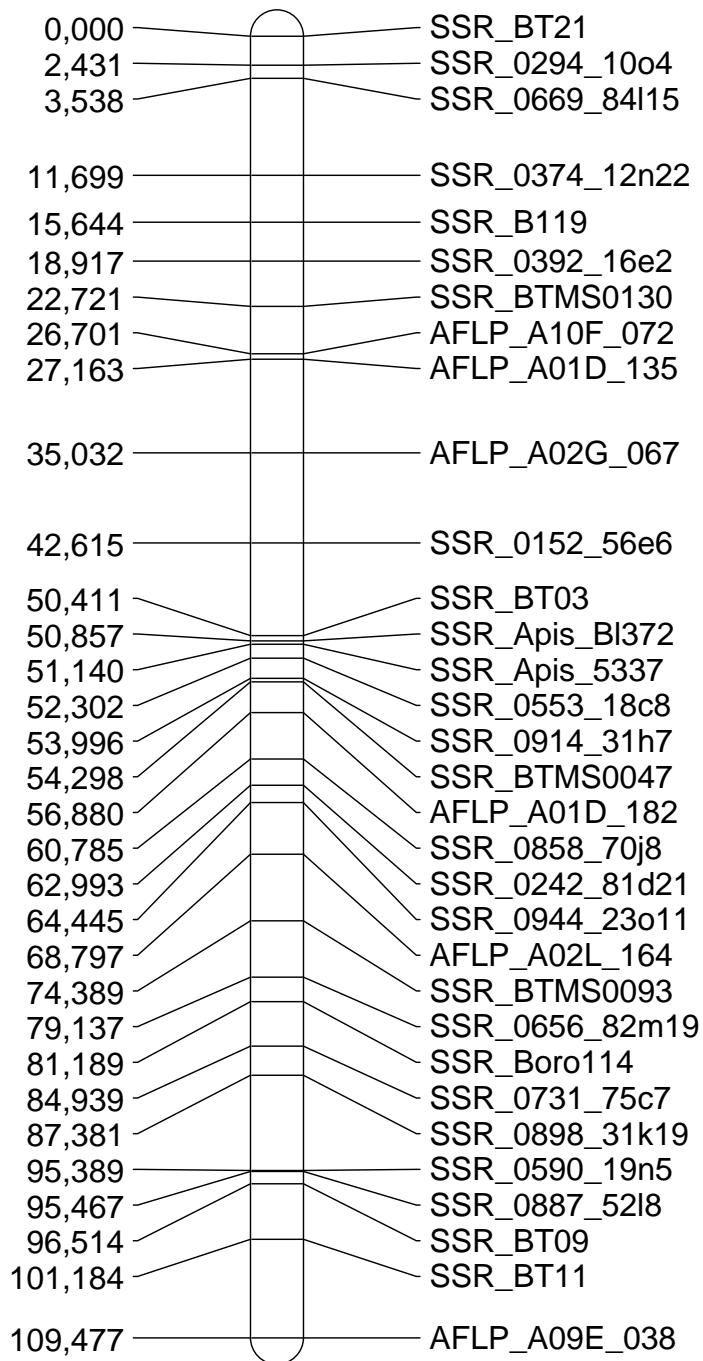

# B10

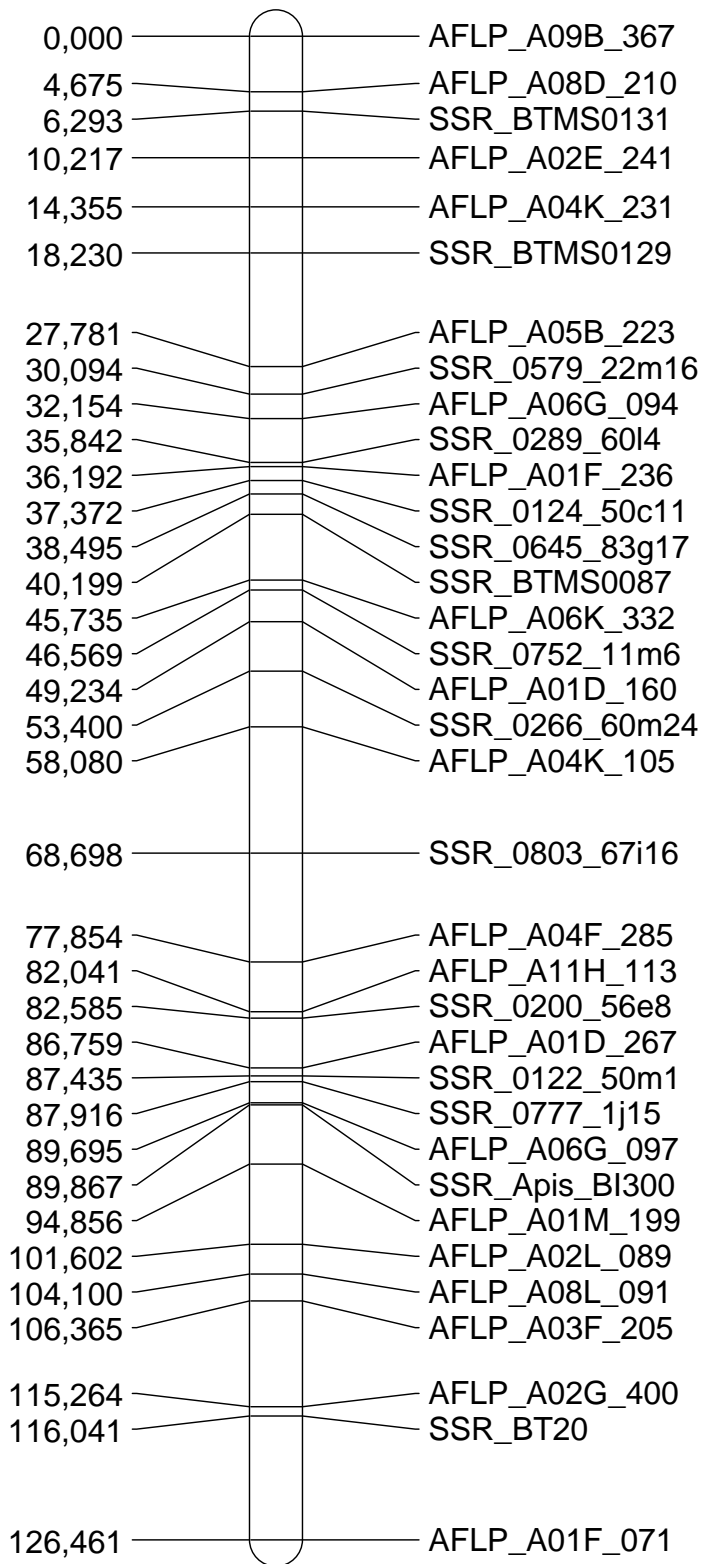

## B11

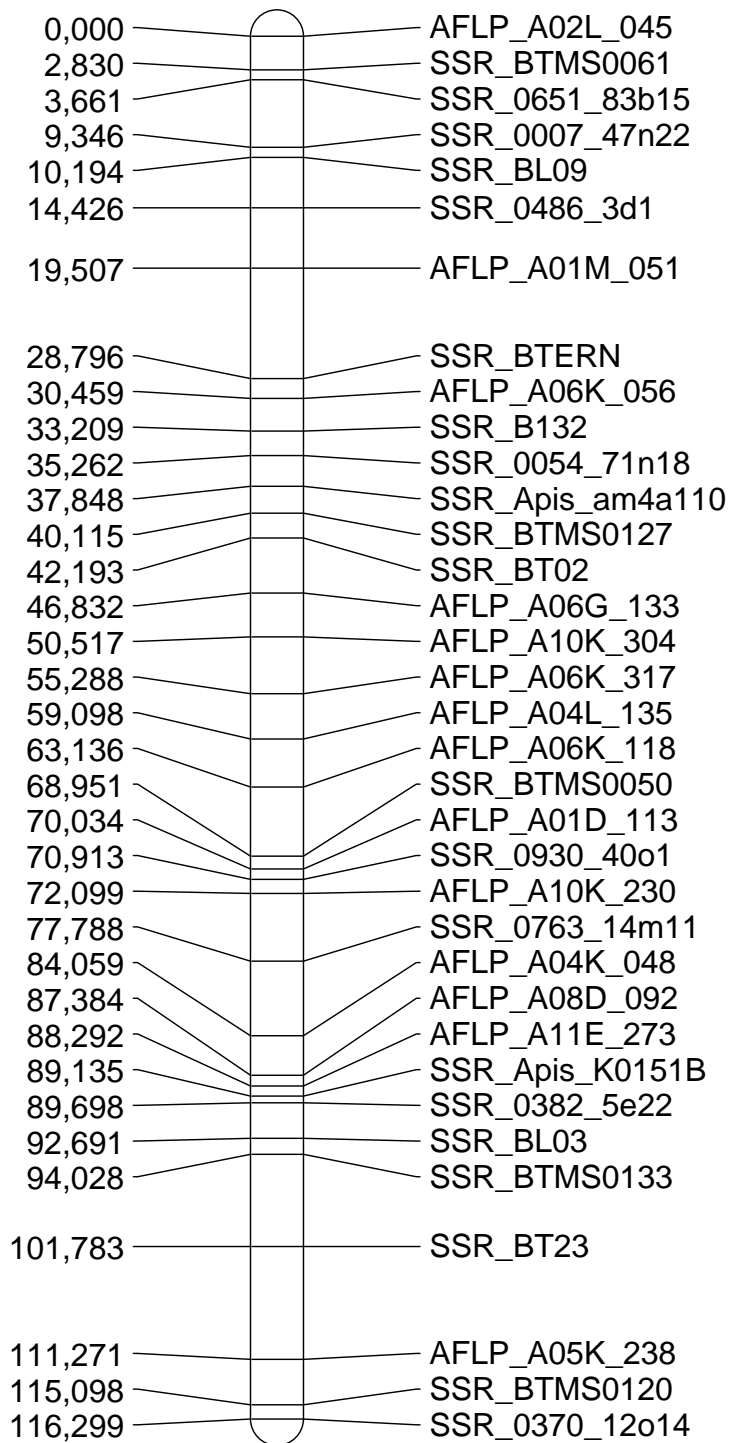

## B12

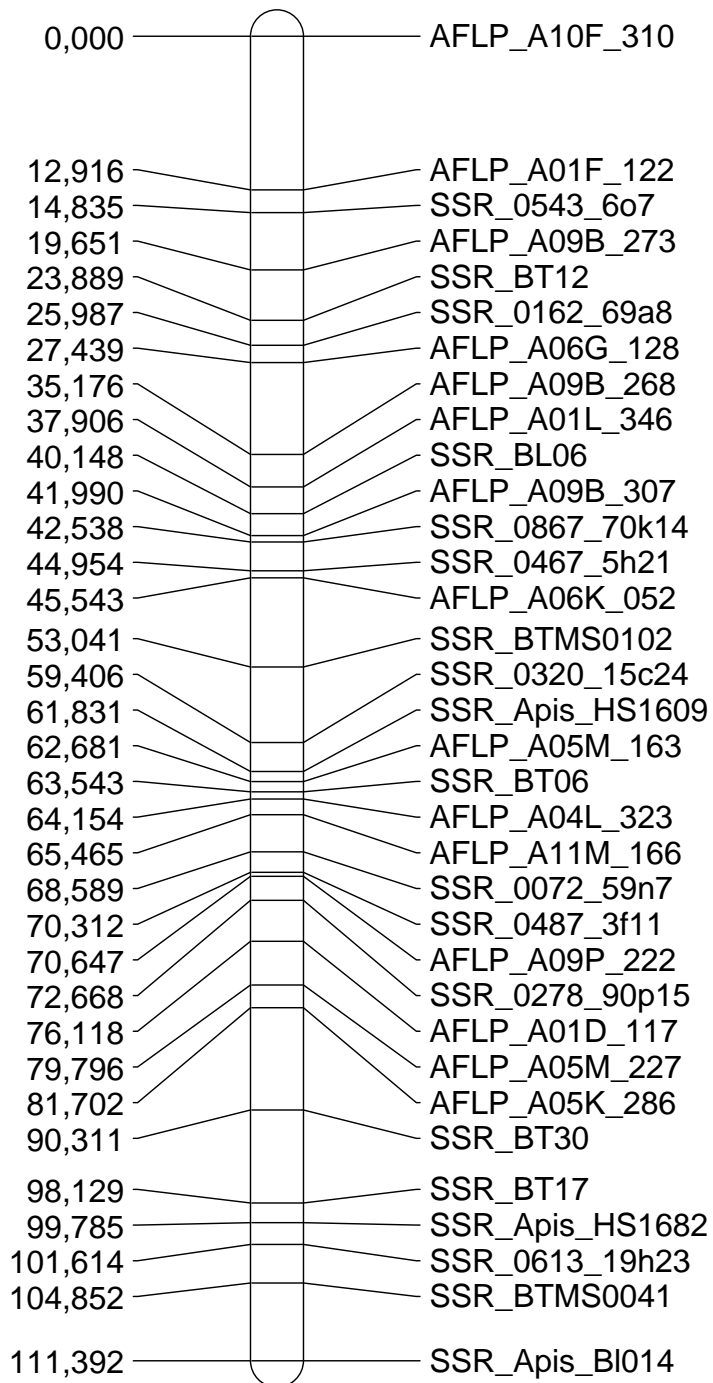

## B13

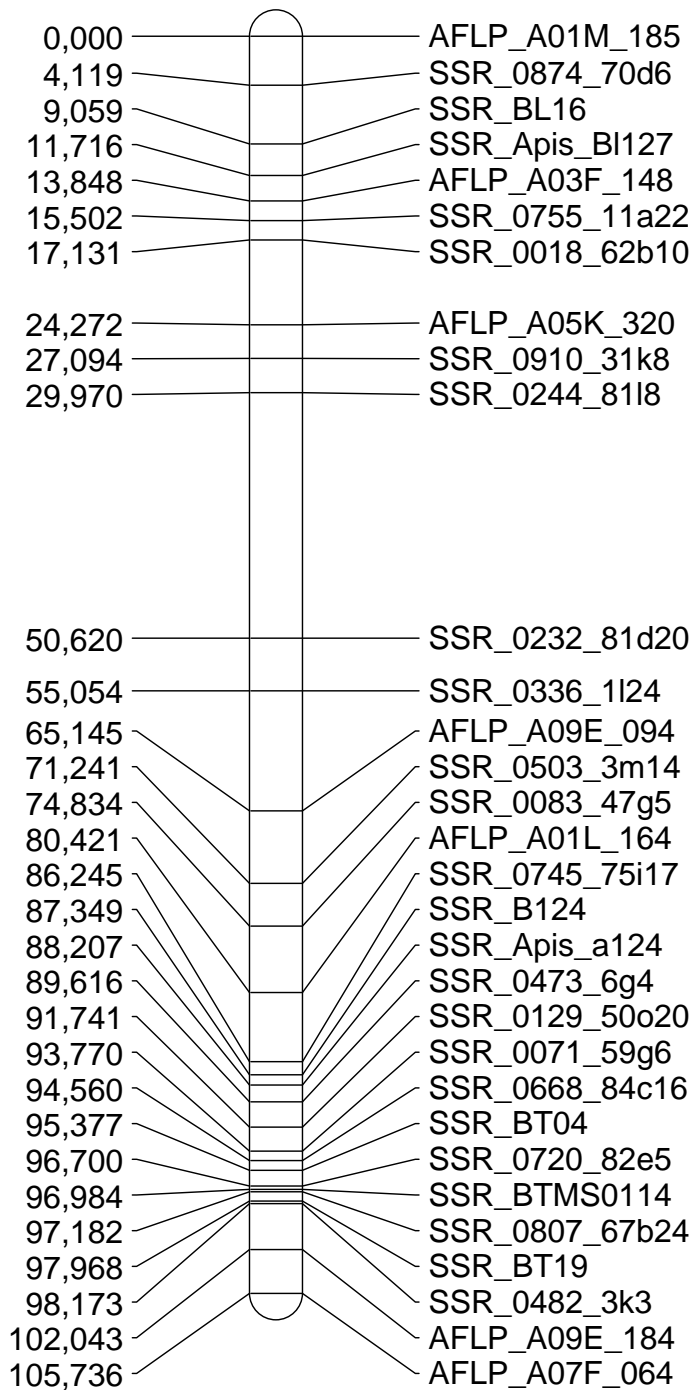

## B14

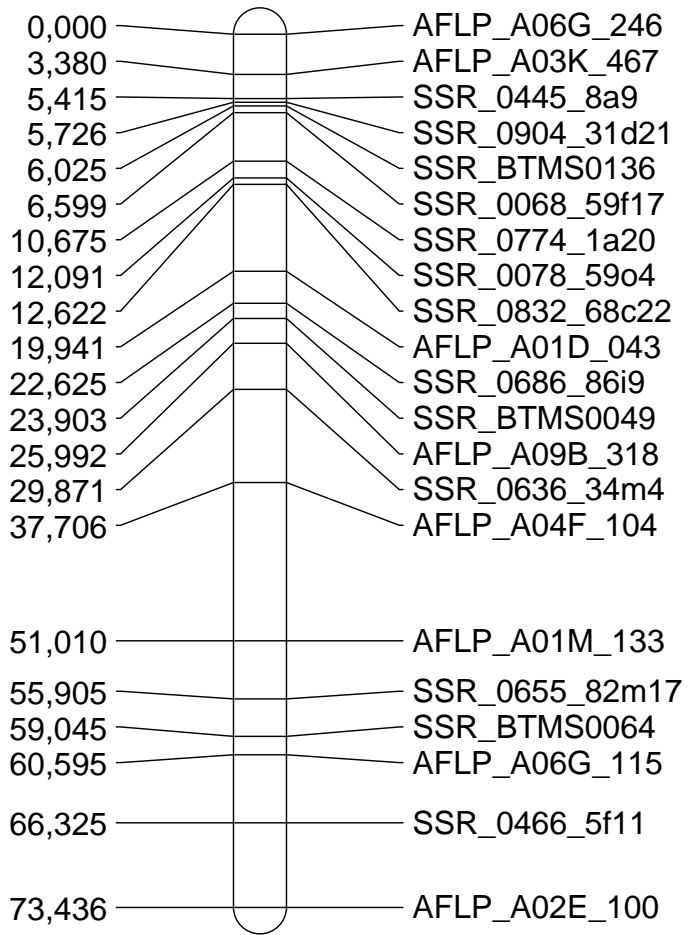

## B15

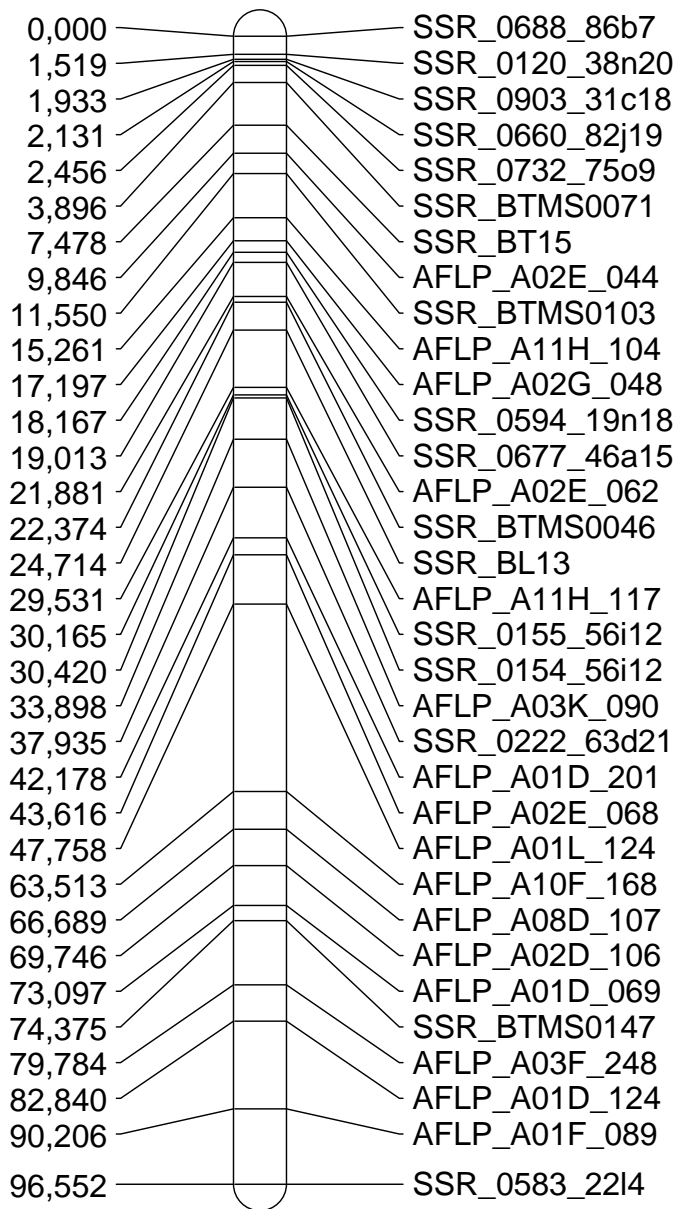

# B16

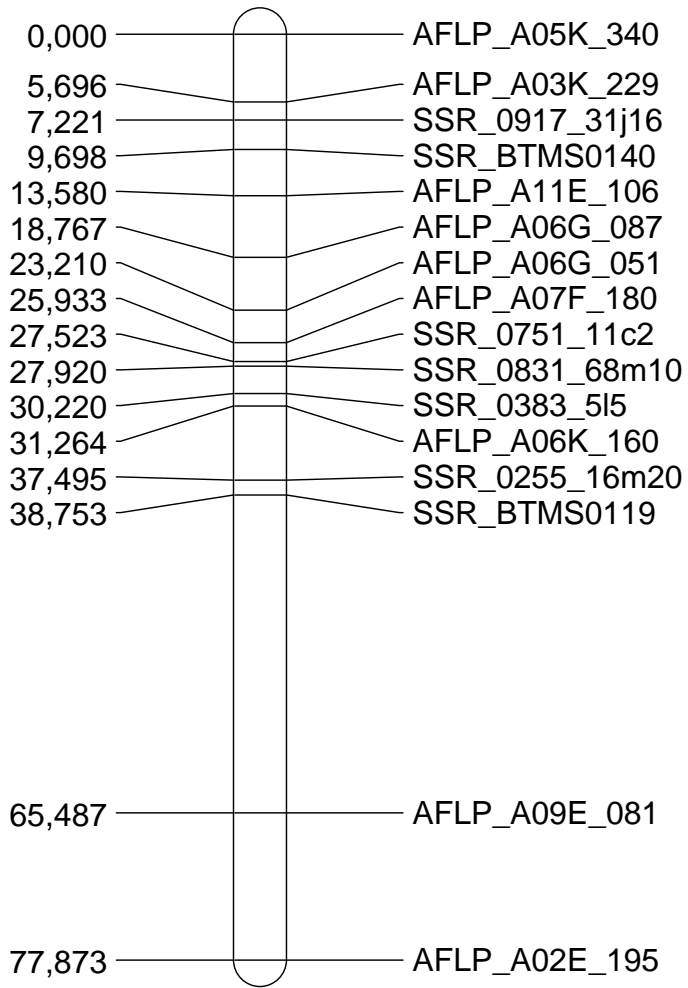

# B17

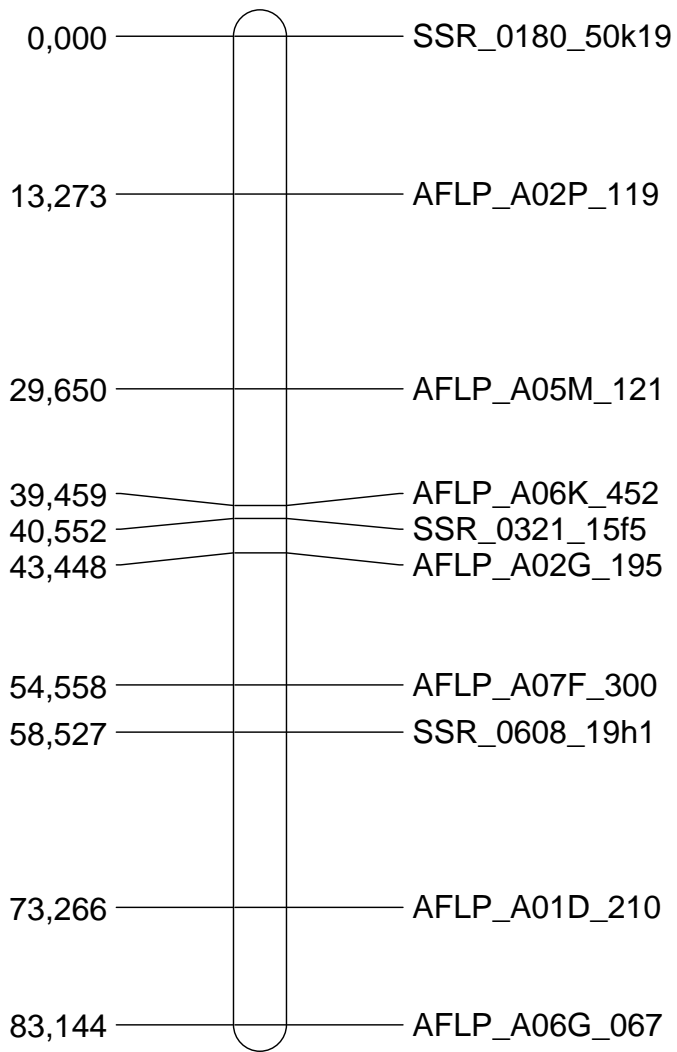

# B18

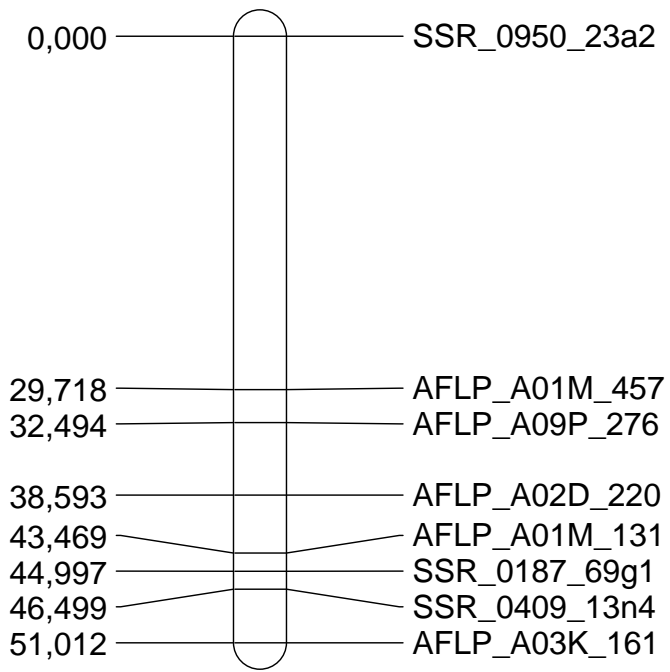

Supplement: Additional file 3 — Bombus terrestris linkage map. This plot shows the Bombus terrestris linkage map with absolute marker positions and marker names for each linkage group. [file 1471-2164-12-48-S3.PDF]
